# Topological defect-propelled swimming of nematic colloids

**DOI:** 10.1126/sciadv.abn8176

**Published:** 2022-08-24

**Authors:** Tianyi Yao, Žiga Kos, Qi Xing Zhang, Yimin Luo, Edward B. Steager, Miha Ravnik, Kathleen J. Stebe

**Affiliations:** ^1^ Chemical and Biomolecular Engineering, University of Pennsylvania, Philadelphia, PA 19104, USA.; ^2^ Faculty of Mathematics and Physics, University of Ljubljana, Jadranska 19, 1000 Ljubljana, Slovenia.; ^3^ Department of Mathematics, Massachusetts Institute of Technology, Cambridge, MA 02139, USA.; ^4^ Department of Chemical Engineering, University of California, Santa Barbara, CA 93106, USA.; ^5^ Mechanical Engineering and Applied Mechanics, University of Pennsylvania, Philadelphia, PA 19104, USA.; ^6^ Condensed Matter Physics Department, J. Stefan Institute, Jamova 39, 1000 Ljubljana, Slovenia.

## Abstract

Topological defects on colloids rotating in nematic liquid crystals form far-from-equilibrium structures that perform complex swim strokes in which the defects periodically extend, depin, and contract. These defect dynamics propel the colloid, generating translation from rotation. The swimmer’s speed and direction are determined by the topological defect’s polarity and extent of elongation. Defect elongation is controlled by a rotating external magnetic field, allowing control over particle trajectories. The swimmers’ translational motion relies on broken symmetries associated with lubrication forces between the colloid and the bounding surfaces, line tensions associated with the elongated defect, and anisotropic viscosities associated with the defect elongation adjacent to the colloid. The scattering or effective pair interaction of these swimmers is highly anisotropic, with polarization-dependent dimer stability and motion that depend strongly on entanglement and sharing of their extended defect structures. This research introduces transient, far-from-equilibrium topological defects as a class of virtual functional structures that generate modalities of motion and interaction.

## Teaser

Nonequilibrium defects on rotated nematic colloids perform swim strokes that propel colloids and generate complex interactions.

## INTRODUCTION

Colloidal propulsion and locomotion, important features of active matter, are produced by diverse mechanisms in natural and synthetic systems (*1*–*6*). Self-propelled swimmers convert chemical energy to generate motion with examples including bacteria and algae (*7*–*9*) that swim by rotation of their flagella, and Marangoni stress–propelled droplets (*10*) that move via gradients in surface stresses and catalytic Janus particles (*11*, *12*) that swim by phoretic motions generated by chemical reaction. Driven colloidal systems rely on external fields, including electrophoretic fields (*13*, *14*), magnetic fields (*15*, *16*), and thermophoretic motion driven by temperature gradients (*17*) to generate colloid translation.

Interactions of swimmers with their environment play important roles in determining their dynamic behavior (*18*, *19*). Hydrodynamic interactions, confinement, and swimmer geometry (*4*, *15*, *20*–*23*) are of central importance in such systems, as they affect locomotion speed and direction and can lead to the formation of dynamic aggregates that can be harnessed as functional structures (*24*, *25*).

Distinctly anisotropic environments like nematic liquid crystals (NLCs) provide means for controlling the microscale locomotion via a combination of their internal orientational order, described by a headless vector field **n** called the director, and their highly anisotropic viscosities. NLC can exhibit topological defects or regions of lost orientational order in the form of lines, points, or even walls that strongly affect the behavior of active nematic colloids (*26*–*31*). For example, bacteria align and move along the local director (*32*), causing them to accumulate at sites of splay and to be depleted from sites of bend (*33*). Self-propelled microdroplets powered by encapsulated bacteria move along paths that can be designed by patterning the NLC director field (*31*).

The anisotropy of nematogens and their orientational order also allow diverse electrokinetic effects that rely on the NLC fluid’s dielectric anisotropy (*34*, *35*). These effects have been harnessed to generate directed colloid motion and complex collective interactions. For example, electrophoretically driven colloids move along or perpendicular to the director field, made possible by the fluid’s dielectric anisotropy (*34*, *35*). Electrophoretically driven motion of Janus beads with metallic hemispheres have been studied to enhance the broken symmetry (*36*, *37*). Electric field–driven flow fields have also been harnessed; electrohydrodynamic rolls oriented within NLC have been exploited to direct the formation and motion of functional assemblies (*38*).

The presence and symmetry of defects on self-propelled colloids and droplets play essential roles in active colloid motion. For example, spherical colloids with dipolar defects move along paths influenced by periodic defect displacement and NLC tilt generated by confinement under AC electric fields (*39*). Defect asymmetry can allow more complex trajectories to emerge. For example, Marangoni stress–propelled NLC droplets lose axial symmetry by flow-induced displacement of their topological defects that generates torques that result in helical trajectories (*40*). This latter example shows the importance of broken symmetry and topological defects in determining nematic colloid behavior and indicates the general importance of dynamic effects in systems with topological defects.

**Fig. 1.**
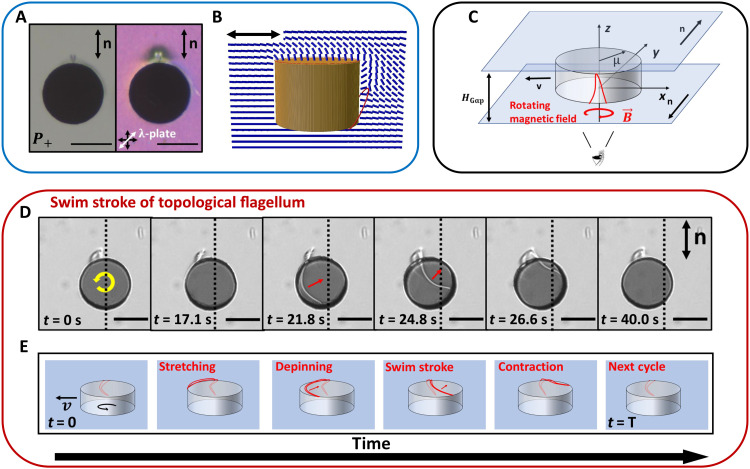
Topological defect’s swims stroke propels colloidal translation and generates far-from-equilibrium interactions. Topological defects seeded on complex rotating colloids restructure under colloid rotation. The rotating colloids translate or swim as their defects perform complex swim strokes whose dynamics propel swimming. Defect elongation breaks symmetry and allows path planning. Far-from-equilibrium pair interactions emerge that enhance or suppress translation. (**A**) Static dipolar defect configuration under bright-field (left) and cross-polarization microscopy with a lambda plate (right). The disk is observed from the bottom in these images. (**B**) Numerical simulation of the static dipolar configuration in a side view showing the nematic director field (blue) and loop-like defect configuration (red). (**C**) Scheme of experimental setup showing a magnetic disk of diameter 2*a* = 75 μm and thickness *H* = 25 μm sandwiched between two glass slides separated by a distance *H*_Gap_ ~ 50 μm with uniform planar anchoring. (**D** and **E**) Time-stamped experimental images (D) and schematics (E) of the swim stroke of a disk colloid under a rotating field with period *T* = 40 s. The vertical dashed lines in (D) indicate the initial position of the center.

In this work, we introduce the concept of swimming propelled by the dynamics of a far-from-equilibrium defect formed on rotating nematic colloids, as illustrated in Fig. 1. Specifically, we study disks with hybrid anchoring in planar cells filled with NLC; a dipolar companion defect loop (Fig. 1, A and B) forms adjacent to the disk, pinned on the disk’s sharp edges. When the disk is rotated by an external magnetic field (Fig. 1C), we observe periodic defect rearrangements; the disclination loop sweeps along the disk’s surface, performing a “swim stroke” that propels the disk in a well-defined direction (Fig. 1, D and E). The translational velocity *v* depends on the angular velocity ω, the sense of rotation, and the defect polarity. These swimmers exhibit complex interactions and form dynamic dimers whose stability depends on their polarization and the topology of their defects.

## RESULTS

Circular disk colloids were fabricated using lithographic methods and made magnetic by sputtering of a Ni film. Thereafter, homeotropic anchoring was imposed on the Ni-coated surfaces, and the disks were released from the substrate and dispersed in 4-cyano-4′-pentylbiphenyl (5CB). The resulting disks have hybrid anchoring, as the face of the disk that is not covered with Ni has degenerate planar anchoring. The colloidal suspension was introduced into the gap between two glass slides with uniform planar anchoring. As is customary in such experiments, these slides are arranged in an antiparallel arrangement to avoid biases in the nematic field by pretilt of the nematogens that can introduced by the process of imposing planar arrangement (*41*–*43*). Disks have typical thicknesses *H* = 25 μm and radii *a* = 37.5 μm; typical gap heights between top and bottom bounding surfaces *H*_Gap_ ~ 50 μm unless otherwise specified. There is some variation in the gap thickness owing to the method of assembling the cell. Last, the cell was placed in a rotating magnetic field generated by a custom-built magnetic control system, and disk rotation is achieved via periodically addressing four electromagnetic coils configured around an inverted optical microscope. An image of the apparatus is provided in fig. S1. Details are described in Materials and Methods.

First, we have simulated equilibrium configurations that correspond to minima of the Landau–de Gennes free energy with a surface potential describing the anchoring of nematic molecules on confining surfaces. These results allow us to understand the director configuration of the system in a static state. Thereafter, we perform simulations in which the system is disturbed from equilibrium. However, because of the complexities of the system dynamics and the scale of the system, we performed numerical simulations of nematodynamics only in the limit in which the director field does not feedback into the hydrodynamics. Time evolution of the tensor order parameter is described by the Beris-Edwards model (*44*). While these simulations allow us to explore the stability of structures and their response to the disk’s rotation, they are not a direct simulation of experiment. The disagreement between experiment and these approximate simulations reveals the importance of backflow effects and defect pinning-depinning dynamics that are not incorporated in the current numerical formulation to the observed disk propulsion. Extensions of simulation to the fully coupled limit that also address near-edge defect pinning and depinning effects essential to the mechanics are the focus of future work. Further information concerning the model and the numerical approach can be found in Materials and Methods.

### Defect configurations around the nematic colloids

When introduced into the NLC-filled planar cell, a complex defect initially forms on the colloid with two disclination loops that connect the top and bottom faces of the disk; these loops appear on either side of the disk arranged in a quadrupolar configuration (fig. S2). Upon perturbation, the defect transforms irreversibly to a static dipolar configuration. Such transitions from metastable quadrupolar configurations to stable dipolar defect configurations are well known for microscale spherical colloids with uniform homeotropic anchoring owing to the differences in elastic free energy related to gradients of the nematic distortion in the domain and the relative costs of the dipolar versus quadrupolar defect (*26*). For such colloids, the quadrupolar configuration is metastable and is observed only under strong confinement in cells of thickness similar to the colloid’s diameter (*45*, *46*). However, unlike homeotropic spheres, the disks feature sharp edges and hybrid anchoring, known to strongly influence defect configurations (*47*, *48*). To probe the stability of the disk’s defect configurations, we study disks in cells with different ratios of gap to disk thickness $\Lambda =\frac{H_{\text{Gap}}}{H}$ for characteristic disk thickness is *H* = 25 μm. The quadrupolar defect configuration is typically observed on all disks immediately after quenching 5CB into the nematic phase. However, after the disks are rotated by the external magnetic field and that field is removed, the probability of observing the quadrupolar mode is reduced. Typically, unless very strongly confined, after perturbation, a dipolar defect forms at either pole aligned with the far field director with equal probability as shown in fig. S3. This suggests that the quadrupolar configuration is metastable and is stabilized by confinement and that the dipolar configuration is the stable state. Given the system’s head to tail symmetry, dipolar defects form at either pole aligned with the far field director with roughly equal frequency. Dipolar defects along the *y* axis at polar angle $\theta =\frac{\pi }{2}$ are termed *P*_+_, whereas those at $\theta =-\frac{\pi }{2}$ are termed *P*_−_.

The dipolar defect configuration features a single loop on one side of the disk (Fig. 1A). Numerical simulation (Fig. 1B) reveals that this defect is a disclination loop anchored at two locations on the degenerate planar face that extends along the side of the disk toward the homeotropic face. This figure shows a side view of the equilibrium configuration of the disk colloid with headless vectors that show the director field winding around the defect. Additional details of the simulation of the equilibrium defect configurations around the disk can be found in figs. S4 and S5. The stable defect configuration is shown schematically in Fig. 1C. Fluorescence confocal polarizing microscopy (FCPM) (*49*) indicates that this disclination loop appears along the *y* axis, which corresponds to the direction of the far field director (fig. S6A). Cross-sectional images in the *xz*- and *xy*-planes (fig. S6, B and C) show that the defect loop extends from the bottom surface toward the top surface adjacent to the disk in agreement with numerical simulations. The dynamics of the dipolar defect configuration upon rotation of the disk generate complex responses with exciting implications.

### Far-from-equilibrium defect propels swimming of individual nematic colloid

**Fig. 2.**
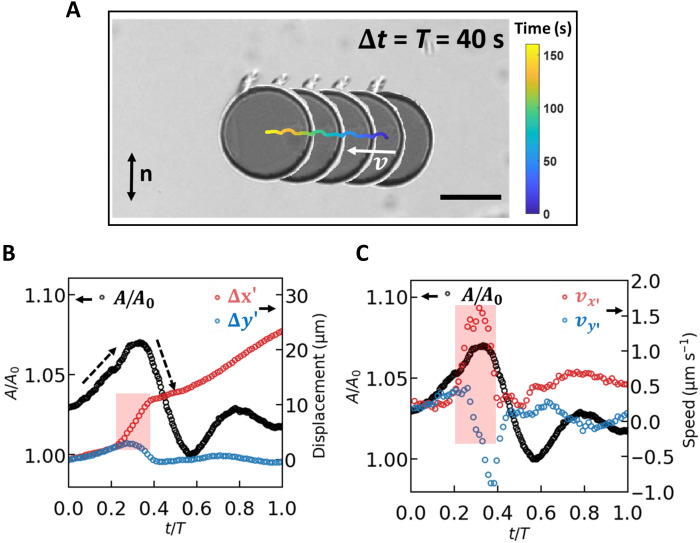
Defect-propelled swimming. (**A**) Superimposed equal time step (Δ*t* = *T* = 40 s) image showing periodic swimming trajectory over four periods of rotation (colored curve). The white arrow indicates the velocity of the disk. Scale bar, 50 μm. (**B**) Normalized projected area *A*/*A*_0_ (black circles, left axis) and displacement parallel (red circles) and perpendicular (blue circles) to the translation direction within one period. The projected area of a tilted disk swimmer (*A*) is always greater than the area of a flat disk swimmer (*A*_0_) due to the finite thickness of the disk colloid. (**C**) Normalized projected area *A*/*A*_0_ [black circles, left axis, repeated from (B)] and translation speed parallel (red circles) and perpendicular (blue circles) to the translation direction within one period.

When the disk is rotated continuously and slowly, the dipolar defect undergoes a periodic rearrangement (Fig. 1, D and E), forming a complex far-from-equilibrium topological structure whose dynamics propel the disk nearly perpendicular to the far field director as shown in Fig. 2A. This propulsion occurs even at small Ericksen number $\text{Er}=\frac{\gamma \omega a^{2}}{K}$, which characterizes the product of the disk rotation frequency ω and the natural time scale for relaxation in the nematic fluid $\tau =\frac{\gamma a^{2}}{K}$ determined by the balance of viscous and nemato-elastic stresses. In these expressions, γ is the rotational viscosity, *a* is the disk radius, *K* is the elastic constant, and ω is determined by the period *T* of the rotating field $\omega =\frac{2\pi }{T}.$ The disclination line initially stretches while remaining pinned on the disk’s sharp edge, storing elastic energy in the form of effective line tension of the defect core and in elastic distortion of the NLC director field that deviates from equilibrium. These effects combined with material flow generate complex torques that cause the disk to tilt, with its projected area (black circles) oscillating twice in each period (Fig. 2B). When the stored energy is high enough to depin the defect, the disclination line contracts by sweeping across the disk’s face and returns to its initial configuration. As this occurs, the disk flattens and subsequently weakly tilts again as the defect reforms its dipolar configuration.

The directed sweeping motion of the far-from-equilibrium disclination line and the tilting motion of the disk play significant roles in generating the broken symmetries that propel the colloid swimmer. As shown in Fig. 2B, roughly half of the swimmer’s displacement occurs in less than one-fifth of the period. During this time, as labeled with pink rectangles in Fig. 2 (B and C), the defect sweeps across the disk’s surface, generating stresses in the thin gap between the disk and the bounding surface. As this occurs, the disk tilts and subsequently flattens. The disk’s projected area allows the tilt angle to be determined (see the Supplementary Materials for details); in this example, the disk reaches a maximum inclination angle of 12° with respect to the horizontal direction. The importance of this sweeping event can also be noted in the speed of the swimmer within one period as shown in Fig. 2C. A sharp peak of swimming speed along the translation direction *v* (red circles) is observed during the defect’s sweeping motion. The defect causes the disk to tilt as it sweeps across the disk’s face. The disk flattens after the defect’s sweeping motion, generating broken symmetries essential to translation. The scallop theorem dictates that nonsymmetric strokes are required to swim in Newtonian fluids in creeping flow. Similarly, in the limit of small Er, nonsymmetric disclination line motion is required to achieve topological swimming in NLC.

To confirm the essential role played by the defect’s swim stroke and to demonstrate that forces from magnetic field gradients cannot produce the observed translation, we have performed a series of control experiments. We have rotated the same magnetic disk colloid in 5CB in the isotropic phase and in the nematic phase in our confined cell. The disk colloid translated with velocities of 0.025 μm/s in the isotropic phase and 0.94 μm/s in the nematic phase under the same rotating external field with period *T* = 20 s. These results confirm that magnetic field gradients over the length scale of the disk are too weak to drive the observed motion. Notably, the velocity in the isotropic phase is decreased although the viscosity is reduced; the viscosity of 5CB decreases with temperature and is lower in the isotropic phase than in the nematic phase. For the above control experiments and for all experiments presented here, the amplitude of the power supply to the coils is 24 V.

Experiments were also performed with a much weaker amplitude voltage of 3 V and, therefore, much weaker magnetic field. In these experiments, the disks were rotated in 5CB in the nematic phase at identical Ericksen numbers. The translation speed of our swimmer under both fields is similar under the same Er (See fig. S7). Because gradients in the experimental domain should scale with the magnitude of the applied voltage, these results also indicate that the swimmer is not driven by the field gradient.

Last, we have performed experiments to confirm the importance of broken symmetries related to the dipolar defect’s dynamics in generating translation. Rotation of disks with a quadrupolar defect translate much slower than the disks with dipolar defect due to reduced broken symmetry. For example, under the same rotating external field with *T* = 60 s, disks with a dipolar defect translate at a speed *v*_dp_ = 0.39 ± 0.06 μm/s while disks with quadrupolar defect translate at *v*_qp_ = 0.08 ± 0.05 μm/s in a Λ ~ 2 cell (see fig. S8). The rotated disks with quadrupolar defects also exhibit periodic disclination line pinning and release (movies S3C and S4). There is a relationship between the distortion of the quadrupolar defect configuration and the disk’s translation speed. Under rotation, some of the quadrupoles become weakly polarized. Those with greater polarization have greater broken symmetry and translate faster. Those that are more symmetric translate more slowly.

We have performed simulations in the fully coupled limit to address the role of backflow on the defect configuration. In simulation, the dipolar configuration is not stable without the director field ansatz at the disk side surface that stabilizes the defect on the disk. Rather, under dynamic perturbation, the dipolar defect transforms to become a quadrupolar defect. Therefore, to quantitatively assess the role of backflow on the defect dynamics, we perform fully coupled simulations of a rotated disk with a quadrupolar defect. We compare these results to simulations performed in the decoupled limit (fig. S9). We show that while backflow changes the strength of the flow (with differences ~8% in this example) and can perturb the defect structure, no defect line pinning emerges. The energetics of defect pinning is a subject of ongoing work.

### Defect elongation, swimming direction, and trajectory planning

**Fig. 3.**
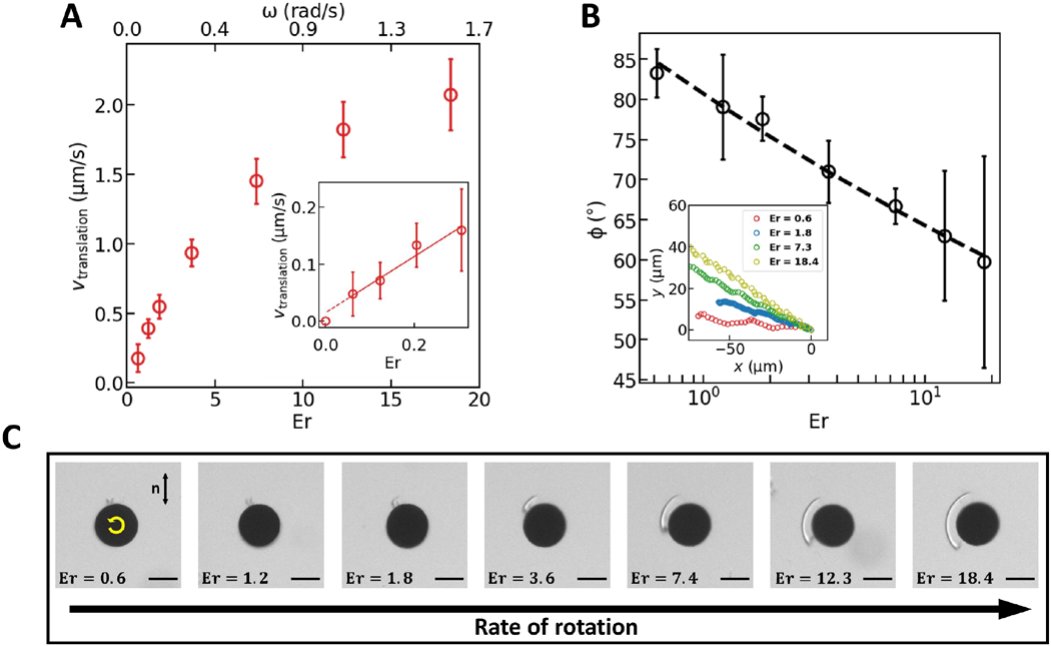
Dynamically elongated defect allows control over swimming direction. (**A**) Translational speed of the defect-propelled nematic swimmer as a function of Er (bottom axis) and average angular velocity ω (top axis). The inset shows the same relationship for small Er. (**B**) Translational direction of the defect-propelled nematic swimmer as a function of Er. The inset shows the trajectories of swimmers under Er = 0.6, 1.8, 7.3, and 18.4, assuming that they have the same initial position at (0,0). (**C**) Defect elongation as a function of Er with the rate of rotation increasing from left to right. Scale bars, 50 μm.

The behavior of these topological swimmers can be characterized in terms of the Ericksen number; changes in swimming behavior at different Er show clearly that the propulsion is affected by both the nematic elasticity and flow. Notably, these effects are substantial even for swimmers moving in creeping flow; the swimmer moves with Reynolds number $\text{Re}<\frac{\rho \omega a^{2}}{\gamma }$ on the order of 10^−5^or less, where ρ is the density of 5CB. For slow rotation, the translational velocity *v* is linear in ω (inset in Fig. 3A) and is directed along an angle ϕ ~ 90°, where ϕ is defined as the angle between the director and the translational velocity. The defect polarity and the disk’s sense of rotation together determine the swimming direction, which can be reversed by changing the disk’s sense of rotation. This reversal of swimming direction is reminiscent of single-flagellated bacteria, which can reverse direction by changing their sense of flagellar rotation (*50*).

As ω increases (finite Er), *v* deviates from the linear relationship, reaching velocities in excess of 2 μm/s for the highest frequencies probed. Moreover, the angle ϕ in Fig. 3B, which characterizes the translation direction, decreases logarithmically with Er. In this regime, highly nonlinear coupling between the flow and director fields, also referred to as the backflow effect, causes the defect to elongate, as shown in Fig. 3C.

**Fig. 4.**
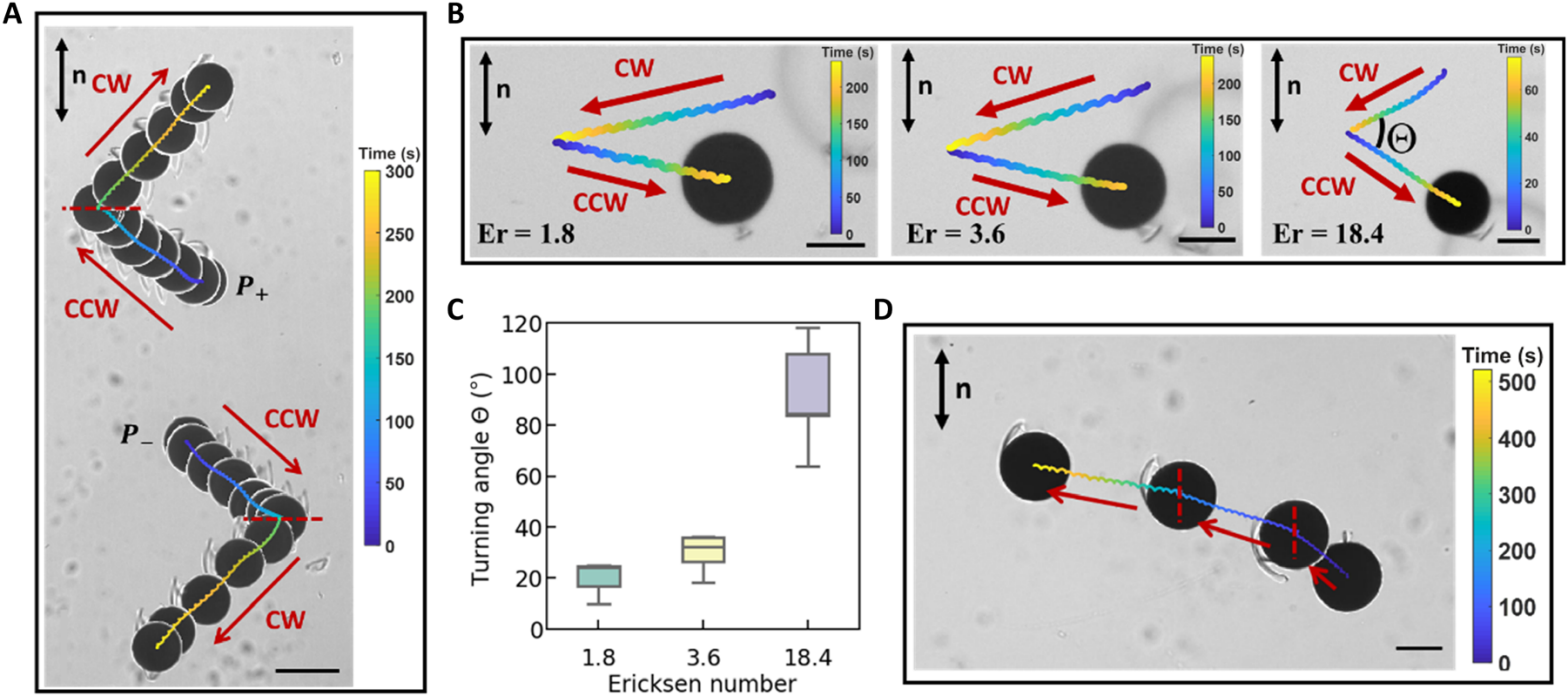
Trajectory planning for the nematic swimmer. (**A**) Superposed images (Δ*t* = 25 s) of two swimmers in same field of view with opposite defect polarity change swimming directions upon reversing sense of rotation of external field. The period of the external field is 4 s, and the red dashed lines indicate the position at which the external field was switched from CCW to CW rotation. The polarities are labeled *P*_+_ and *P*_−_, respectively, adjacent to the disk’s initial position. (**B**) Reversing the sense of rotation of single swimmers from CW to CCW rotation with Er = 1.8, 3.6, and 18.4 (left to right). The colored curves indicate the trajectories of swimmers. (**C**) Turning angles Θ of single swimmer when their senses of rotation are reversed with different Er. The turning angle Θ is defined as in (B). (**D**) Superposed images of a curved trajectory of a swimmer following a curved trajectory in 520 s. The period of the external field is changed from 4 to 12 s and then 12 to 36 s at the locations indicated by the red dashed lines. Scale bars, 100 μm (A) and 50 μm (B and D).

Defect elongation adjacent to the disk further breaks the symmetry of the system and biases the swim direction, as zones of reduced nematic order have lower effective viscosity. The degree of defect elongation depends on defect depinning from the rough, sharp edges of the disk, which has a stochastic character, explaining the broad range of ϕ observed for different disks at a given Er. Consider a *P*_+_ dipole, under counterclockwise (CCW) rotation, the defect on a disk elongates primarily in the quadrant defined by $\frac{3\pi }{2}\leq \theta \leq 2\pi$. This generates a zone of reduced effective viscosity, causing the disk to swim in the direction of the elongated defect along a path that forms an angle $\frac{\pi }{2}-\phi$ with respect to the −*x* axis. Under clockwise (CW) rotation, this same disk’s defect elongates in the quadrant $0\leq \theta \leq \frac{\pi }{2}$, causing the disk to swim along a path with angle $\frac{\pi }{2}-\phi$ with respect to the *x* axis. The dependence of swimming direction on Er gives rise to additional control for trajectory planning. Swimmers move along a path and, upon reversal of the sense of rotation, follow trajectories that are mirror symmetric with respect to the *x* axis (Fig. 4, A and B) with some turning angle Θ = π − 2ϕ.

Furthermore, at any value of Er, owing to the symmetries of the system and the fact that the nematic director is a headless vector, colloids with opposite defect polarities migrate in opposite directions under the same external field. Figure 4A illustrates both the mirror symmetric trajectories and the motion of colloids with opposite polarities in opposite directions. Two disks with opposite defect polarity at *t* = 0 s move in opposite directions (red arrows) under the same external field. When the sense of disk rotation is reversed by changing the external field from CCW to CW rotation (indicated by the red dashed lines in Fig. 4A), the swimmers execute V-shaped trajectories. Further experimentation shows that, as expected, the turning angle increases with Er (Fig. 4, B and C). However, these responses are also affected by the stochastic character of the defect elongation, causing different disks to follow a broad range of turning angles at any given Er (Fig. 4C).

Last, the dependence of translation direction on Er can be exploited to steer the swimmer during rotation; the defect-propelled colloid followed a curved path by tuning the rate of rotation of the swimmer at locations indicated by the red dashed lines as shown in Fig. 4D. By exploiting these dependencies, a disk colloid with given defect polarity can explore a half space by simply tuning the sense of rotation and frequency of the external field.

### Scaling analysis of the propulsion mechanisms

We attribute the observed swimming behavior to mechanisms associated with the presence of the topological defect and the associated anisotropic order within the NLC. Here, we characterize the relevant mechanisms in terms of the disk size, viscoelastic properties of the nematic, and disk rotation frequency to estimate the swim velocities that each mechanism can produce.

We first consider the effects of elastic stress exerted on confined objects in a nematic medium, which is described by a stress tensor formulated by Ericksen (*42*). The elastic stress displaces objects to minimize the nematic elastic free energy. Assuming that the length scale over which the director distortion occurs is of the order of disk radius *a*, the elastic stress scales as $\sigma_{K}∼\frac{K}{a^{2}}$. The associated elastic force is calculated by integrating the elastic stress over the disk surface, which scales as 2π*a*^2^ + 2π*aH* where the disk’s thickness *H* ~ 2/3*a*. The resulting propulsion force is therefore of the order of $F_{K}∼\frac{10\pi K}{3}$. Another approach to estimate the elastic force on the disk is through the concept of defect line tension. Geometric frustration due to the rotation of the disk generates a periodic reshaping of a pinned defect line. During this swim stroke, the pinned defect line pulls on the colloidal particle. The elongated pinned defect line acts on the disk surface with a force π*K*/4 ln (*a*/*a*_core_) (*51*), where we have again used the radius *a* as a measure of the length scale and *a*_core_ is the size of defect line cores (for 5CB, *a*_core_is of the order of a few nanometers). As the logarithmic factor changes very slowly with *a* and the result for the experimental radius is similar in magnitude to the *F_K_* above, we shall use the scaling of $\frac{10\pi K}{3}$ in our analysis. At equilibrium, elastic forces act symmetrically on the disk and therefore generate no propulsion. As the nematic configuration is periodically driven out of equilibrium at low Er, the disclination line elongates quasistatically, depins, and contracts at the rate determined by the balance of elasticity and viscosity. The periodic reshaping of the disclination line leads to elastic stresses acting on the disk surface, and the corresponding upper estimate of the propulsion speed can be found

$$v\sim \frac{F_{K}}{C_{D}}\sim \frac{10\pi K}{3C_{D}}$$ (1)

where *C*_D_ is the drag coefficient of the disk translating between two plates. For a disk translating in a confined environment between two parallel plates, the drag coefficient is estimated from lubrication theory as $\frac{C_{D}}{\eta a}\sim \frac{4a}{h_{0}}+\frac{4a}{h_{1}}$, where η is the estimated average viscosity of 5CB, *h*_0_ and *h*_1_ are the gap thickness between the disk surface and the bottom plate and the top plate, respectively. For a typical experiment, FCPM reveals that $h_{0}\sim \frac{1}{2}h_{1}\sim 8.6\;\mu m$. By defining a dimensionless parameter $\varepsilon =\frac{h_{0}}{2a}$, the drag coefficient can be estimated as $C_{D}=\eta a\frac{3}{\varepsilon }$. Using these expressions for *F*_prop_ and *C*_D_, and relevant parameter values of *K* = 6.5 pN (*51*), *a* = 37.5 μm, and η = 0.064 Pa s (*51*), we predict a translational velocity *v* ~ 108 μm/s. Note that, while the disk rotation frequency is not explicitly included in Eq. 1, it enters through the periodic forcing of the elastic deformation.

We next consider the lubrication forces associated with the flow generated by the defect’s sweeping motion. The disk periodically tilts and flattens as the disclination line elongates and sweeps across its surface. The resulting flow results in a hydrodynamic force in the thin gaps between the disk and the bounding plates. On the top face of the disk, where the defect performing the swim stroke is absent, the disk does not experience a net force due to the scallop theorem because the tilting and flattening are completely reversible. However, below the disk, as the defect sweeps over the bottom face, it causes the disk to tilt. The disk then flattens after the defect has left the gap. The resulting difference in viscosity in the thin gap with and without the defect avoids the constraints of the scallop theorem and results in a net hydrodynamic force on the disk.

To estimate this hydrodynamic force, we assume the thickness of the liquid film absent tilt between the disk and the bottom wall *h*_0_ is small compared to the disk radius *a*, consistent with the experiment. Defining the small parameter $\varepsilon =\frac{h_{0}}{a}<<1$, we expand Stoke’s equation for an incompressible fluid to find the thin film equations (*52*, *53*). We integrate these equations to find the pressure field exerted on the disk and integrate the pressure field to find the hydrodynamic force. Details are given in the Supplementary Materials.

We consider 1 cycle of disk tilting and flattening generated by the defect’s sweeping motion; the tilting and flattening events each occur over time 0.3 *T*. The disk tilts with $\overset{\cdot }{\alpha }>0$ in the presence of the defect between the disk’s surface and the bottom confining wall. The disk flattens with $\overset{\cdot }{\alpha }<0$ after the defect has left this thin gap. When the defect is present, the film has an effective viscosity η_defect_. When the defect is absent, the film has the viscosity representative of bulk nematic η. We approximate the disk as a plate of width and length given by twice the disk radius *a*. Defining ρ_0_ = αε^−1^, the *x*-directed force is $F=-\frac{6a\overset{\cdot }{\alpha }}{\alpha^{3}}\;h_{0}\eta *(\;\text{ln}\frac{\left(2+\rho \;\right)}{\left(2-\rho \;\right)}(12-\rho^{2})-12\rho )$where η^*^ is either η_defect_ or η depending on whether the defect is present or absent. Using this expression, the velocity over the cycle can be estimated

$$v\approx \frac{1}{C_{D}}(F_{\text{defect}}+F_{\text{no-defect}})=(\frac{a\eta }{C_{D}})\frac{6\;h_{0}|\overset{\cdot }{\alpha }|}{\alpha^{3}}(\frac{\Delta \eta }{\eta })(\;\text{ln}\frac{\left(2+\rho \;\right)}{\left(2-\rho \;\right)}(12-\rho^{2})-12\rho )$$ (2)

where $\frac{\Delta \eta }{\eta }=\frac{\eta -\eta_{\text{defect}}}{\eta }$ falls in the range of 0.01 to 1 (*31*). For the swim stroke reported in Fig. 2, *T* = 40 s and the average tilt angle during the tilting event is α_avg_ = 6.0^°^ ≈ 0.105. The rate of disk tilting can be estimated $\overset{\cdot }{\alpha }=\alpha_{\text{max}}/(0.3\;T)\approx 8.7\times 10^{-3}s^{-1}$. Using these values and the aforementioned drag coefficient, we estimate the translational velocity in the *x* direction $v=2.50\frac{\Delta \eta }{\eta }\;\mu m/s$. This indicates that the translation speed during this part of the swim stroke will be in the range of ~0.02 to 2.50 μm/s depending on the viscosity ratio. The expansion of the Stoke’s equations to find the thin film equations was performed for finite tilt angle comparable to the small parameter, i.e., α ∼ ε. The resulting force expression is singular in the limit as α tends to zero. However, to consider the behavior for α << ε, the expansion can be repeated and the singularity in α would be relieved.

Last, we consider the anisotropic viscous stresses on either side of the disk owing to the presence of the elongated defect adjacent to the disk. As the disk rotates in the nematic fluid, it experiences a viscous force that is dependent on the structure of the nematic field. The side of the disk with the nematic defect present is a region of different and, in principle, lower (*40*), effective viscosity than the opposite side of the disk. Such a difference in viscosities can explain the behavior of spinning disks at higher Er in which the elongated defect structure determines the direction of the swimmer. To estimate the magnitude of propulsion velocity due to this anisotropy in viscous stresses, we assume that the effective viscosity on the side that contains the defect to be η_1, side_ while that absent the defect is assumed to be η_2, side_. The shear at the disk side wall of height *H* is estimated as *v*_sidewall_ = ω*a*. The shear force density on the side wall therefore equals *dF*_1_/*dS* = η_1, side_ω in the region with the defect. Integrating over *dS* = *aHd*θ, we obtain $F_{1}=\int_{0}^{\pi }d\theta \mathrm{ah}{\omega \eta }_{1,\text{side}}\text{sin}\theta =2\mathrm{ah}{\omega \eta }_{1,\text{side}}$. The net force on the disk can then be written as $F\sim F_{1}-F_{2}=2\mathrm{ah}\omega \eta \frac{\Delta \eta_{\text{side}}}{\eta },$ where $\frac{\Delta \eta_{\text{side}}}{\eta }=\frac{\eta_{1,\text{side}}-\eta_{2,\text{side}}}{\eta }\approx \frac{\Delta \eta }{\eta }$. The net force generates a disk translational velocity of

$$v\sim \frac{2H\omega }{C_{D}}\frac{\Delta \eta }{\eta }$$ (3)

For experimental parameters at ω = 1 s^−1^, this estimate yields a velocity in the range ~0.02 to 2 μm/s.

These estimates provide an upper bound on the propulsion mechanism. The estimation of the velocity spans two orders of magnitude due to the uncertainty of the term $\frac{\Delta \eta }{\eta }$. This value, to our knowledge, cannot be accurately specified, especially for far-from-equilibrium systems like ours. Therefore, we used the range 0.01 to 1 commonly cited in the literature (*31*) for thermotropic nematics (*51*, *54*).

There are interesting open issues associated with the defect-driven swimming. For example, the elongation and subsequent instability of the disclination line along the rotating disk’s edge, captured both in experiments (Fig. 1, D and E) and simulation in the decoupled limit (fig. S10 and movie S5), play a prominent role in the swimming phenomenon. Edge roughness may also play a role promoting disclination line pinning (fig. S12).

However, a comparison of the simulation and the experiment shows important differences that might be attributed to differences in scale and to missing physics in the current code. In simulation, the defect on the rotated disk has a quadrupolar character, which might be attributed to the differing scale of simulation and experiment. The disclination line does not elongate as significantly along the disks’ edge, which might be attributed to the need to include mechanisms for defect pinning/depinning along rough edges that remain to be elucidated. Last, in simulation, the disks do not tilt or translate. In the current simulation, the flow does not feedback into the elastic energy landscape, and forces on the colloid are not extracted from the stress tensor evaluated on the surface of the colloid. These differences underscore the importance of such effects, which will be explored in future work.

To conclude, we have considered two forces that contribute to the swimming motion and a mechanism to explain the manner in which the elongated defect can bias swimming direction. These include elastic forces owing to gradients in the nematic director field, lubrication forces in the thin film between the disk and bounding surfaces, and anisotropic viscous forces on either side of the disk, respectively. Scaling analyses of the three propulsion mechanisms generate estimates for the swimming speed like that observed in the experiment. It is likely that all three effects contribute. This is not unexpected, as all mechanisms are due to pushing the nematic configuration out of equilibrium.

### Confinement

**Fig. 5.**
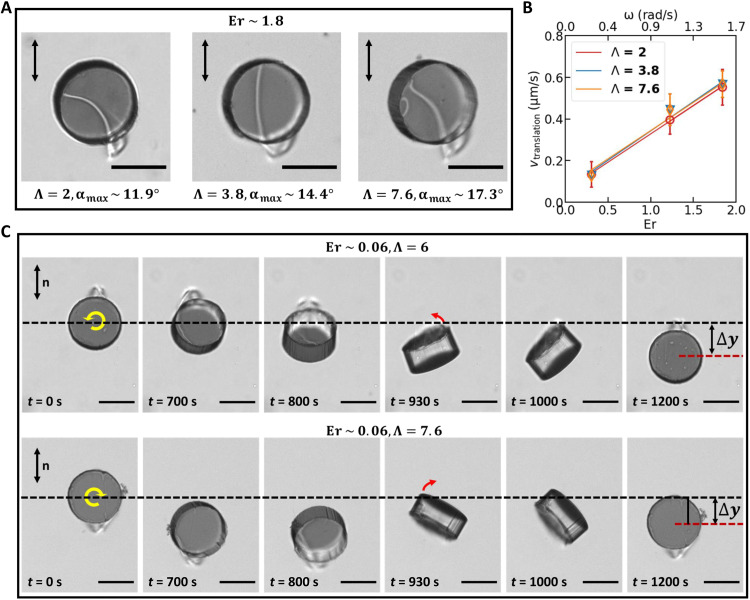
Confinement effects. (**A**) Disks rotated in cells with ratio of gap height to disk thickness over the range 2 ≤ Λ ≤ 7.6 at *Er* = 1.8 have slightly increased tilt angles. (**B**) Disks rotated in cells with ratio of gap height to disk thickness over the range 2 ≤ Λ ≤ 7.6 show similar dependence on Er. (**C**) Slow rotation of weakly confined disks generates a tilting/flopping motion underscoring the importance of confinement in limiting disk tilting. Tilting and rotation of an individual swimmer weakly confined cells under slowly rotating CCW (top row, Λ = 6) and CW (bottom row, Λ = 7.6) external rotating field (*T* = 1200 s; *Er* = 0.06).

Analysis suggests that the swimming modality depends crucially on elastic stresses and lubrication forces under the disk as the defect performs its swim stroke. To probe the role of confinement in this swimming modality, we have studied our swimmer in cells of different 2 ≤ Λ ≤ 7.6. For fixed Er = 1.8, weaker confinement allows the disks to tilt to slightly larger angles as shown in Fig. 5A, while the thickness of the film beneath the disk remains on the order of ~10 μm. The translational speed is similar among the disks (fig. S13). Furthermore, for 0.3 ≤ Er ≤ 1.8, the swimmers move with similar translational speed as shown in Fig. 5B. This supports the concepts advanced in the scaling analysis that the elasto- and hydro-dynamics in the thin film beneath the disk play a prominent role and the dynamics in the film above the disk is of lesser importance.

At very slow rotation and very weak confinement, however (e.g., Er = 0.06; Λ = 6 and Λ = 7.6), the defect’s swim stroke is lost. Rather, the disk’s motion is highly complex, tilting onto its side with α ~ 90^∘^ and flopping down, translating as it does so, as shown in Fig. 5C and movie S6. As this occurs, the defect becomes unstable and drives complex three-dimensional (3D) motions of the disk. This behavior is akin to that explored previously in the literature in which anisotropic particles forced to rotate in NLCs display shape-dependent responses, as their companion defects become topologically unstable and rearrange with complex colloidal dynamics (*55*–*58*). These observations underscore the importance of confinement in limiting the disk’s tilt to weak slopes.

### Dynamic defect interactions determine dynamic dimer stability

**Fig. 6.**
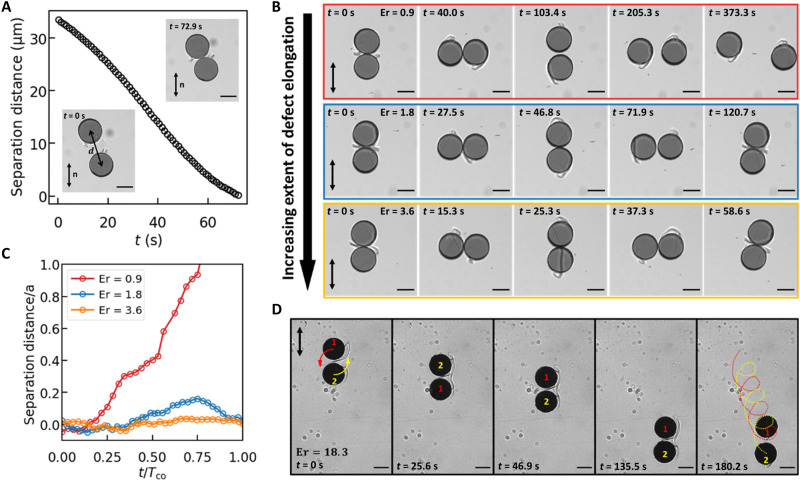
Pair interactions between swimmers. (**A**) Change in interparticle distance (*d*-2a) by quasistatic nematic interaction for colloids with opposite defect polarity absent rotation. Insets show configurations at *t* = 0 s and the final antiparallel arrangement at 72.9 s. (**B**) Time-stamped images of dynamic pair interactions of colloids with opposite defect polarities under rotating fields of *T* = 80 s (top row), 40 s (middle row), and 20 s (bottom row), respectively. (**C**) Normalized separation distances as a function of time of Er = 0.9 (red), 1.8 (blue), and 3.6 (orange). (**D**) Time-stamped images of corotation and translation of dimer formed by two colloids (labeled as 1 and 2) with similar defect polarity under rotating field of *T* = 4 s. The red and yellow dash curves in the last frame indicated the helical trajectories of colloids 1 and 2, respectively. Scale bars, 50 μm.

We present a discussion of pairs and multibody interactions to show the importance of far-from-equilibrium defects in these interactions. Pair interactions among swimmers depend strongly on the relative polarity of the two swimmers and the topology of their defects. At rest, two swimmers with opposite polarity self-assemble in an antiparallel manner (Fig. 6A) similar to their spherical counterparts with homeotropic anchoring (*59*). Swimming introduces dynamic interactions. The stability of dynamic dimers of opposite polarity depends strongly on rotation rate, as shown in Fig. 6 (B and C) and movie S7. In Fig. 6, we show representative behavior for a pair of disks rotated at different frequencies. At Er ~ 0.9, the two swimmers corotate as a dimer for a short period of time, separate, and move away from each other (first row in Fig. 6B and red circles in Fig. 6C). For faster rotation (Er~1.8), the swimmers reform a bonded dimer after a small gap is created during the corotation (second row in Fig. 6B and blue circles in Fig. 6C). We hypothesize that such distinct dynamic interactions result from the interplay of repulsive interaction between splay distortions from the disk’s curved boundaries and attractive defect-defect interactions. Further increases in Er above a threshold lead to stable dimer corotation (bottom row, Fig. 6B; separation distance is indicated by orange circles in Fig. 6C). For these rotation rates, the defects become elongated enough to entangle and merge to cement stable dimers. The antisymmetric arrangement of the individual disks in dimers formed by swimmers of opposite polarity promotes stable rotation without significant translation. For example, an individual colloid swims at an average velocity of 0.55 μm/s, while a dimer translates at 0.031 μm/s under the same field at Er = 1.8.

Disk-to-disk variation affects these dynamics and thresholds. For example, we study a pair of disks that corotate about each other with complex dynamics, separating, and rejoining, with period for their complex motions *T*_CO_ typically greater than the disk rotation period for 0.3 ≤ Er ≤ 1.8 in a Λ = 2 cell (fig. S14A). Similar results are found for more weakly confined disks, with Λ = 3.8 (fig. S14B). We also report a pair of disks that form a stable dimer at Er = 3.6 but migrate apart for Er = 1.8 in Λ = 2 cell (fig. S14C).

Disk-to-disk variation in defect elongation can also strongly affect the stable dimer rotational dynamics. For example, in movie S8, two dimers formed by disks of opposite defect polarities rotate at very different speeds under an external rotating field of period *T* = 6 s. While the two swimmers in the lower left dimer corotate at a faster rate of 34.2 s per period, the other dimer on the right rotates nearly three times slower, at a rate of 113.0 s per period. We attribute this difference in rotational dynamics to the different extents of defect elongation, as one of the swimmers in the slower dimer has a very weakly elongated defect in comparison to the others. For our individual swimmers, there is a stochastic element to the defect elongation, which relies on pinning on, depinning from, and elongation of the defect adjacent to the disk’s random rough sharp edges. These differences in elongation alter the dimer formation and rotational dynamics as well.

For disks of similar polarity, dimer formation also depends strongly on Er that determines defect topology. Disks with distinct, separate elongated defects comigrate in the same direction without forming a dimer (movie S9). However, above some threshold, the defects of the two disks merge to form a complex shared structure holding the two swimmers together with enhanced broken symmetry; the dimers rotate and translate, as shown in Fig. 6D and movie S10, with an average speed of 2.07 μm/s, similar to that of an individual swimmer under the same external field. As swimmers within such dimers orbit their partner, they follow helical trajectories as shown in the last frame of Fig. 6D. The observed corotation period for these dimers of 45.7 s ± 1.8 s is far longer than the driving field *T* = 4 s. In all cases, the dimers rotate with angular velocities far slower than the rate of rotation of the external field.

**Fig. 7.**
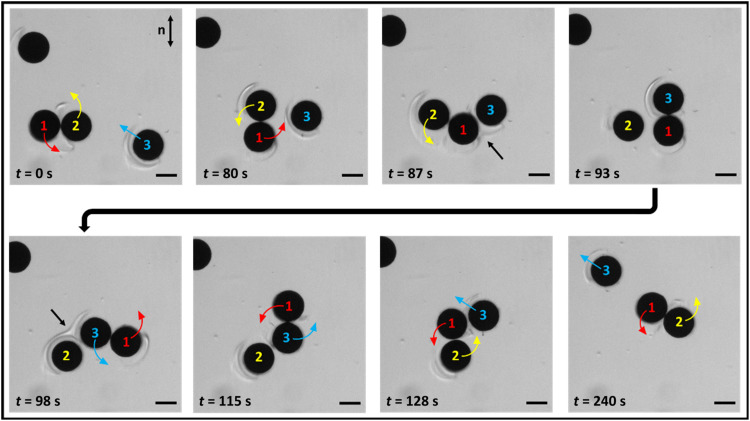
Multibody interactions. Time-stamped images of dynamic interactions between three swimmers (labeled as 1, 2, and 3) under an external CCW rotating field of *T* = 4 s. The arrows of different colors indicate instantaneous velocities of the swimmers, respectively. Black arrows point to the region where significant defect interactions occur. Scale bars, 50 μm.

Last, multibody interactions occur that rely on dynamic interaction between the extended defects of multiple swimmers (movie S8). For example, the formation and breakdown of an unstable trimer have been observed, as shown in Fig. 7, where a third swimmer (labeled as 3) enters a stable dimer formed by two swimmers (labeled as 1 and 2), interacts with both swimmers via their extended defects, and eventually leaves the dimer and proceeds independently. Systematic study of pair, multibody, and collective interactions will be the focus of future research.

## DISCUSSION

In this work, we introduce topological defect-propelled swimming of nematic colloids. We develop rotating magnetic disk colloids with complex, elongated defects that perform a swim stroke that drives their translation. Geometric frustration dominates for small rotation rates, and the colloid’s speed is linear in Er. At faster rotation rates, significant defect elongation allows the swimming direction to be tuned for path planning. These defect-propelled swimmers exhibit far-from-equilibrium pair interactions that differ significantly from their static dipolar counterparts.

The swimming of our nematic colloid and its reliance on far-from-equilibrium defect dynamics make our system highly distinct from systems of spherical paramagnetic and ferromagnetic colloids in isotropic fluids. Typically, the colloids in these systems are rotated about an axis in the plane of the confining cell, so that they exhibit rolling (*16*, *60*–*62*). These rollers display rich interactions and intriguing collective dynamics including synchronization and the formation of unstable fronts. We expect that collective dynamics will emerge in our system as well. The dependence of dimer dynamics in our system on far-from-equilibrium defect dynamics suggests that collective behavior will also depend on defect elongation and entanglement thresholds. This is a focus of future research.

The field of active colloids is developing in tandem with the fields of microrobotics and reconfigurable materials. The defect-driven motion of colloidal microswimmers could be harnessed in this context (*63*), to exploit defect-driven microswimmers that interact with passive colloidal cargo to build functional structures.

In conclusion, defect-propelled swimming of nematic colloids opens opportunities for soft material manipulation and unveils open, exciting questions. For example, our disks have sharp edges and hybrid anchoring conditions that generate defects that are not clearly defined by the Poincaré-Hopf or Gauss-Bonnet theorem that relates uniform anchoring to required topological charge (*48*, *64*). Under quasistatic rotation, the defect elongates along the disk’s edge and subsequently depins from the edge; the physics that regulate these transitions and their relationship to colloid geometry are unexplored. Under finite Er rotation, the dipolar defect elongates in the flow field by a dynamic instability. Our colloids also form shared, dynamically changing defects that merge and separate, subject to topological transitions whose rules are far from evident. Last, we have reported dynamic dipolar interactions for nematic colloids, introducing a spinner colloid with significantly rate-dependent interactions. We have shown that dynamic defect interactions play primary roles in pair interactions and multibody effects. Future work will address dynamics of systems of pairs, trimers, and many swimmers to probe collective phenomena including the formation of reconfigurable motile structure (*16*, *60*, *65*), front propagation, and stability (*16*, *66*).

## MATERIALS AND METHODS

### Fabrication of ferromagnetic disk colloids and assembly of NLC cell

Circular disk colloids of diameter 2*a* = 75 μm and thickness *H* = 25 μm were fabricated out of SU-8 photoresist (Kayaku Advanced Materials Inc.) following standard lithographic processes on a supporting wafer. Thereafter, a layer of Ni was sputtered onto the surface using a Lesker PVD75 DC/RF Sputterer to make the colloids ferromagnetic. Subsequently, treatment with 3 weight % (wt %) solution of *N*-dimethyl-n-octadecyl-3-aminopropyl-trimethoxysilyl chloride (Sigma-Aldrich) imposed homeotropic anchoring conditions on the disk’s Ni-coated surfaces. The treated disk colloids were then released from the wafer and dispersed in 5CB (Kingston Chemicals). Glass slides were spin-coated with polyimide (PI-2555, HD Microsystems) and rubbed with a velvet cloth along the desired direction to impose uniform planar anchoring. To form cells with gap heights *H*_Gap_ ~ 50 μm, two glass slides with uniform planar anchoring were assembled in an antiparallel fashion and glued together using a ultraviolet (UV)–sensitive epoxy with two layers of 15 μm plastic spacers in between. To avoid any artifacts from pretilt (tilting away from an angle parallel to the surface) of the nematogen, the rubbed slides are typically assembled in antiparallel arrangements in experiments in planar cells (*41*–*43*). This reduces bend deformations in the domain that might arise from this effect. To fabricate cells with larger gap widths, spacers of different height were used to configure the cells. Last, a suspension of disk colloids in 5CB was introduced into the cell by capillarity in the isotropic state of 5CB. Depending on the thickness of the nickel layer, the coated disk could either appear transparent (nickel layer, ~20 nm) or black (nickel layer, ~200 nm). While the transparent disk allowed us to visualize the swim stroke across the surface of the disk, colloids with thicker coating have stronger magnetic moments, enabling faster rates of rotation.

### Controlled rotation of disk colloids

To rotate the magnetic disk colloids, the assembled NLC cell was placed in a rotating magnetic field generated by a custom-built magnetic control system. The system consists of two orthogonal pairs of electromagnetic coils (APW Company) mounted on an aluminum supporting structure arranged around the workspace. Visual feedback was provided by a charge-coupled device camera (Point Grey Grasshopper3 Monochrome) mounted on a Zeiss inverted microscope (ZEISS Axio Vert.A1). Each coil pair was powered independently using a programmable power supply (XG 850W, Sorensen) whose outputs were controlled by a data acquisition board (USB-3104, Measurement Computing) and a Python algorithm written in-house. Sinusoidal time-dependent voltages of identical amplitudes are applied on each pair, and the waveforms are separated by a π/2 phase lag to achieve a circularly rotating field whose periods varied from 4 to 1200 s for this study. The schematics and picture of the setup are shown in fig. S6.

### Characterization of static dipolar defect using FCPM

The configuration and location of the defect in a dipolar configuration around a disk colloid along the vertical *z* axis were determined by FCPM (*49*). The NLC, 5CB, was doped with an anisotropic dye *N*,*N*′-Bis(2,5-di-tert-butylphenyl)-3,4,9,10-preylenedicarboximide (Sigma-Aldrich) at 0.01 wt %. At such low concentration, dye molecules coalign with the NLC molecules while preserving the properties of 5CB and fluoresce when aligned parallel to the polarization direction of the excitation light. FCPM images of the disk colloid with a dipolar defect in a planar NLC cell were obtained using an inverted IX81 Olympus microscope equipped with an FV300 Olympus confocal scan box. A polarizer was placed between the sample and the objective to rotate the polarization of the scanning laser.

### Characterization of surface roughness of the disk colloids using atomic force microscopy

The surface roughness of the top and side surfaces of the disk colloid after Ni deposition was characterized using a Bruker Icon atomic force microscopy in standard tapping mode. Characterization of a 10 μm by 10 μm area gave a root mean square roughness *R*_q_ = 3.29 nm with a peak value of 107 nm, indicating that the top surface is nanoscopically smooth with a few isolated rough sites in the hundred nanometer range as shown in fig. S12 (A and B). The roughness of the side surface was obtained by placing the disk on its side, adhered to a planar support. The root mean square roughness *R*_q_ of a 3 μm by 3 μm scanning area (fig. S12, C and D) was 19.4 nm with a peak value of 213 nm. The side surface has greater roughness than the top surface. We attribute such surface roughness to nonuniform Ni deposition and typical resolutions achieved in 2D UV photolithography. These rough sides may facilitate the pinning of the disclination line. Note that the roughness on the sides and edges are similar in size to the defect core ~10 nm.

### Numerical simulations

We performed numerical simulations using a Q-tensor order parameter description of nematodynamics. The director field **n** is obtained as a main eigenvector of the Q-tensor and the degree of order *S* as its main eigenvalue. Equilibrium structures correspond to a minimum of the free energy

$$\begin{array}{cc} F= & \int_{\text{bulk}}dV[\frac{A}{2}Q_{\mathrm{ij}}Q_{\mathrm{ji}}+\frac{B}{3}Q_{\mathrm{ij}}Q_{\mathrm{jk}}Q_{\mathrm{ki}}+\frac{C}{4}{\left(Q_{\mathrm{ij}}Q_{\mathrm{ji}}\right)}^{2}+\frac{L}{2}(\partial_{k}Q_{\mathrm{ji}})(Q_{k}Q_{\mathrm{ij}})]+\\ & \int_{\text{disk bottom surf}.}d\mathrm{SW}\;{\left({\tilde{Q}}_{\mathrm{ij}}-{\tilde{Q}}_{\mathrm{ij}}^{⊥}\right)}^{2} \end{array}$$ (4)

which is calculated from the bulk and the surface contributions. The parameters *A*, *B*, and *C* are nematic phase parameters that dictate the degree of order in the equilibrium homogeneous director field *S*_eq_. The quantity *L* is the tensorial elastic constant and is directly proportional to the director elastic constant *K*. The surface integral is performed only over the disk bottom surface, where the anchoring of nematic molecules is planar degenerate. On the disk side walls, the disk top surface and the cell’s top and bottom boundaries, the director field is fixed. The planar-degenerate surface is modeled by a Fournier-Galatola potential (*67*), where ${\tilde{Q}}_{\mathrm{ij}}=Q_{\mathrm{ij}}+\frac{S_{\text{eq}}}{2}\delta_{\mathrm{ij}}$, ${\tilde{Q}}_{\mathrm{ij}}^{⊥}=(\delta_{\mathrm{ik}}-\nu_{i}\nu_{k}){\tilde{Q}}_{\mathrm{kl}}(\delta_{\mathrm{lj}}-\nu_{l}\nu_{j})$, and $\vec{\nu }$ is the surface normal.

Q-tensor dynamics are described by the Beris-Edwards model (*51*)

$${\overset{\cdot }{Q}}_{\mathrm{ij}}=\Gamma H_{\mathrm{ij}}+S_{\mathrm{ij}}$$ (5)

where the dot represents the advective time derivative, *H_ij_* is the molecular field driving the nematic orientation toward a free energy minimum

$$H_{\mathrm{ij}}=-\frac{1}{2}(\frac{\delta F}{\delta Q_{\mathrm{ij}}}+\frac{\delta F}{\delta Q_{\mathrm{ji}}})+\frac{1}{3}\frac{\delta F}{\delta Q_{\mathrm{kk}}}\delta_{\mathrm{ij}}$$ (6)

and Γ is the rotational viscosity parameter. *S_ij_* describes the nematic response to flow gradients

$$S_{\mathrm{ij}}=(\zeta A_{\mathrm{ik}}-\Omega_{\mathrm{ik}})(Q_{\mathrm{kj}}+\frac{\delta_{\mathrm{kj}}}{3})+(Q_{\mathrm{ik}}+\frac{\delta_{\mathrm{ik}}}{3})(\zeta A_{\mathrm{kj}}+\Omega_{\mathrm{kj}})-2\zeta (Q_{\mathrm{ij}}+\frac{\delta_{\mathrm{ij}}}{3})Q_{\mathrm{kl}}\partial_{l}v_{k}$$ (7)

where $\vec{v}$ is the flow field, *A_ij_* = (*∂_i_v_j_* + *∂_j_v_i_*)/2, Ω*_ij_* = (*∂_i_v_j_* − *∂_j_v_i_*)/2, and ζ is the nematic alignment parameter. On the planar degenerate surface, the Q-tensor follows the dynamics of

$${\overset{\cdot }{Q}}_{\mathrm{ij}}^{\text{surf}}=\Gamma_{\text{surf}}[\frac{1}{2}(H_{\mathrm{ij}}^{\text{surf}}+H_{\mathrm{ji}}^{\text{surf}})-\frac{1}{3}\delta_{\mathrm{ij}}H_{\mathrm{kk}}^{\text{surf}}]$$ (8)

where Γ_surf_ is the surface rotational viscosity parameter, and

$$H_{\mathrm{ij}}^{\text{surf}}=-\frac{\partial f_{\text{vol}}}{\partial (\partial_{k}Q_{\mathrm{ij}})}\nu_{k}-\frac{\partial f_{\text{surf}}}{\partial Q_{\mathrm{ij}}}$$ (9)

is the surface molecular field, calculated from the bulk and surface free energy density given by Eq. 4.

Simulations in which the disk does not rotate were solved for the fluid at rest. For simulations with a rotating disk, the flow field was calculated by a lattice Boltzmann method with a moving boundary condition, and the resulting stationary flow field was used in Eq. 5. Simulations were performed using a finite difference method to solve Eq. 5. The simulation in fig. S5 was obtained on a 580 × 580 × 140 mesh size with disk radius *a* = 105Δ*x* and disk height *h* = 70Δ*x* (where Δ*x*is the mesh resolution), while other simulations were performed on a 380 × 380 × 280 mesh with *a* = 95Δ*x* and *h* = 140Δ*x*. A plane with a no-slip velocity boundary condition and a fixed planar director field are used at the top and at the bottom of the simulation box. Periodic boundary conditions are used in the lateral directions of the numerical simulation box. The mesh resolution is set as $\Delta x=1.5\xi_{N}=1.5\sqrt{L/(A+{\mathrm{BS}}_{\text{eq}}+\frac{9}{2}{\mathrm{CS}}_{\text{eq}}^{2})}$, where ξ_N_ is the nematic correlation length that sets the size of the defect cores. The following values of the model parameters are used:ζ = 1, *B*/*A* = 12.3, *C*/*A* = −10.1, *W* = 0.5*L*/Δ*x*, Γ_surf_ = Γ/Δ*x* (unless otherwise specified), and a time step of 0.1 (Δ*x*)^2^/(Γ *L*) (*68*). The results of the simulations are expressed using the mesh resolution Δ*x*, rotational viscosity parameter Γ, and elastic constant *L*.

Numerical simulations that minimize the Landau–de Gennes free energy in terms of the Q-tensor order parameter yield the 3D director field. In these simulations, the mesh size cannot exceed the defect core size set by the nematic correlation length, which is orders of magnitude smaller than the particle size in experiment. The presence of this length scale requires that simulations be run in dimensional form. Such simulations are typically limited to colloids of ~1 μm in diameter owing to computational cost. A complicating factor is that colloids of different sizes have different stable defect configurations. For example, for homeotropic spheres in aligned nematic, dipolar defects are stable for particles of micrometer size, while the quadrupolar configuration is stable for submicron unconfined particles. At the micrometer scale, quadrupolar configurations are metastable states stabilized by confinement. For disks with hybrid anchoring, at the scale of the simulations, quadrupolar configurations are favored over dipolar configurations, whereas dipolar configurations are favored in the experiment performed with 75-μm-diameter disks. In this setting, it is not yet known where the transition in stability occurs.
