# Supplementary material for: Topological defect-propelled swimming of nematic colloids
Source: Sci Adv. 2022 Aug 24;8(34):eabn8176. doi: 10.1126/sciadv.abn8176 (PMC10939095; doi:10.1126/sciadv.abn8176)
Supplement: Supplementary file 1 — Supplementary text Figs. S1 to S14 [file sciadv.abn8176_sm.pdf]

Science Advances
AAAS

Supplementary Materials for

Topological defect-propelled swimming of nematic colloids

Tianyi Yao *et al.*

Corresponding author: Kathleen J. Stebe, kstebe@seas.upenn.edu

Sci. Adv. **8**, eabn8176 (2022)
DOI: 10.1126/sciadv.abn8176

The PDF file includes:

Supplementary text
Figs. S1 to S14
Legends for movies S1 to S11

Other Supplementary Material for this manuscript includes the following:

Movies S1 to S11

Supplementary Text and Figures

S1. Experimental Apparatus

The experimental apparatus consists of a system of four electromagnetic coils configured around the stage of an optical microscope. Details of the manner in which the coils are addressed, and the rotating field is controlled are provided in the Materials and Methods section in the main text.

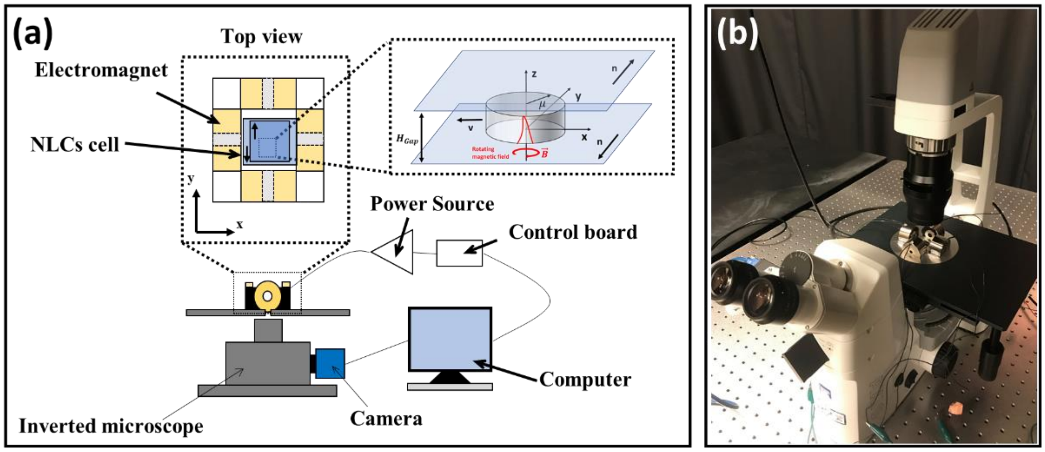

Fig. S1. Experimental setup. Schematics (a) and picture (b) of the experimental apparatus.

S2 Defect configurations of the hybrid disk colloid

Disk colloids form companion defects in both quadrupolar and dipolar modes. We performed experiments to image these defects, and to probe their relative stability after perturbation, as explained in the main text. The static hybrid disk colloid in the quadrupolar mode is shown in experiment and simulation in Fig. S2.

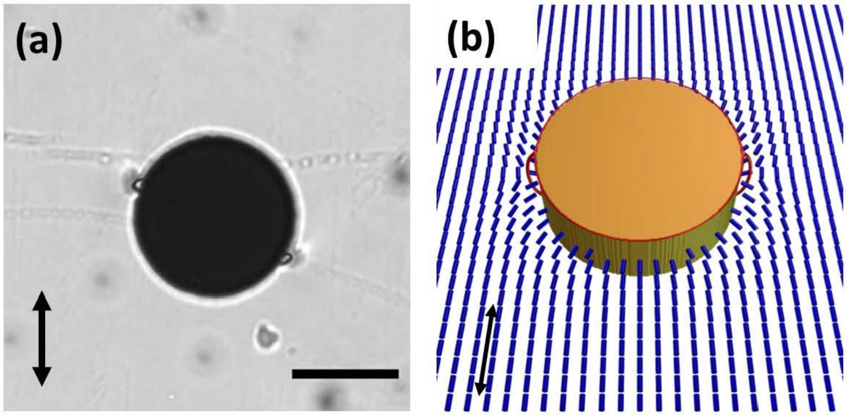

Fig. S2. Quadrupolar defect structure on the static hybrid disk colloid. Microscopic image (a) and numerical simulation (b) showing the quadrupolar defect configuration around a disk colloid. The double-headed arrows indicate the far-field director. Scale bar is $50\mu m$.

We studied the relative stability of the two defect configurations on the hybrid disk colloids. The details are given in the main text. The results in Fig. S3. indicate that, in experiment, the quadrupolar defect is metastable, while the dipole is stable.

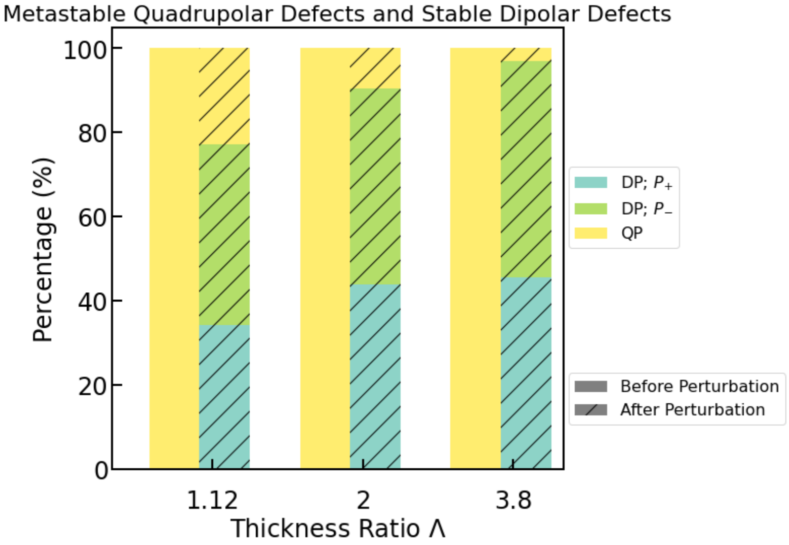

Fig. S3. The quadrupolar defect configuration is metastable while the dipolar defect configuration is the stable state. Statistics for single swimmers' defect configurations before and after rotation under the magnetic field as a function of confinement. 'DP' and 'QP' in the legend denote dipolar and quadrupolar defect configurations, respectively. P_+ and P_- indicate the polarization of the dipolar defect along the y-axis as defined in the main text. In the histogram, the left column indicates that all defects are in a quadrupolar mode before perturbation. The right column indicated the distribution of dipolar and quadrupolar modes after perturbation. To construct this graph, the number of observations N for each confinement ratio was $N_{\Lambda=1.12}=35$; $N_{\Lambda=2}=114$; $N_{\Lambda=3.8}=33$.

Stabilization of dipolar configuration is a challenge at the scale of the simulation, which is limited by computational cost. We have performed numerical experiments to stabilize the dipolar versus quadrupolar configurations around the disk colloid to allow us to understand qualitative features of the free energy landscape and of the director fields in the vicinity of the colloid. The dipolar solution is only metastable for specific side-wall director profiles. Higher disk aspect ratios compared to experiments are used to promote the stability of the dipolar structure, since it is known that the stability of a dipolar solution is increased for large colloidal particles compared to the nematic correlation length (ref. 26 in the main text). Conditions were identified that allowed dipolar defect loops to be realized in simulation. These simulated anchoring conditions explored in simulation to stabilize the dipolar configuration were not explored in experiment. In Figure S4 we test how different director profiles on the disk's sidewall affect the dipolar solution. When the

director field on the side wall is taken to be completely perpendicular to the surface, the dipolar solution is unstable and in time transforms into a quadrupolar field (Fig. S5).

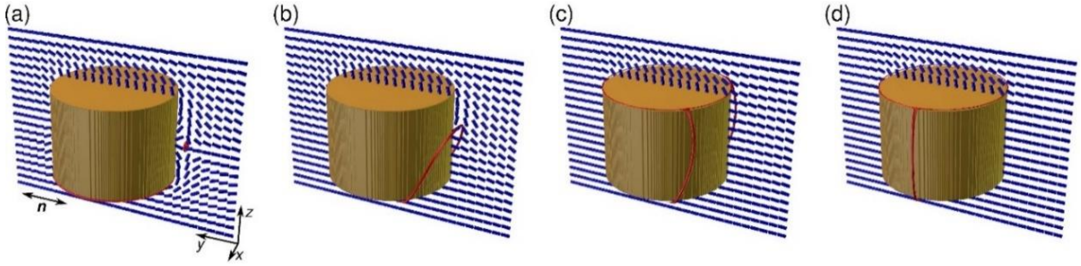

Fig. S4. Defect configurations on static hybrid disks in numerical simulations. Depending on the boundary condition on the disk surface, simulations can show various dipolar or quadrupolar solutions. In all panels, anchoring on the disk’s bottom surface is planar degenerate and its top surface is homeotropic. The disk is positioned in a cell with the director orientation along y axis on the top and the bottom plane. (a) On the sidewall of the disk a splay-like director field is defined, going from $-z$ to $+z$ orientation between the bottom and the top surface. This boundary condition stabilizes a point defect in the form of a small loop along the director far-field axis. (b) If the splay-like boundary condition is enforced only on the top half of the disk's sidewall, a defect line is the stable dipolar configuration. The defect line is pinned at two points to the bottom edge of the disk, and its cross-section shows a half-integer profile. (c) If the boundary condition on the whole sidewall is homeotropic, the nematic field evolves from the initial dipolar ansatz into a solution with two defect lines, each pinned to the top and the bottom edge. This dipolar solution is unstable and in time evolves into the quadrupolar field (d).

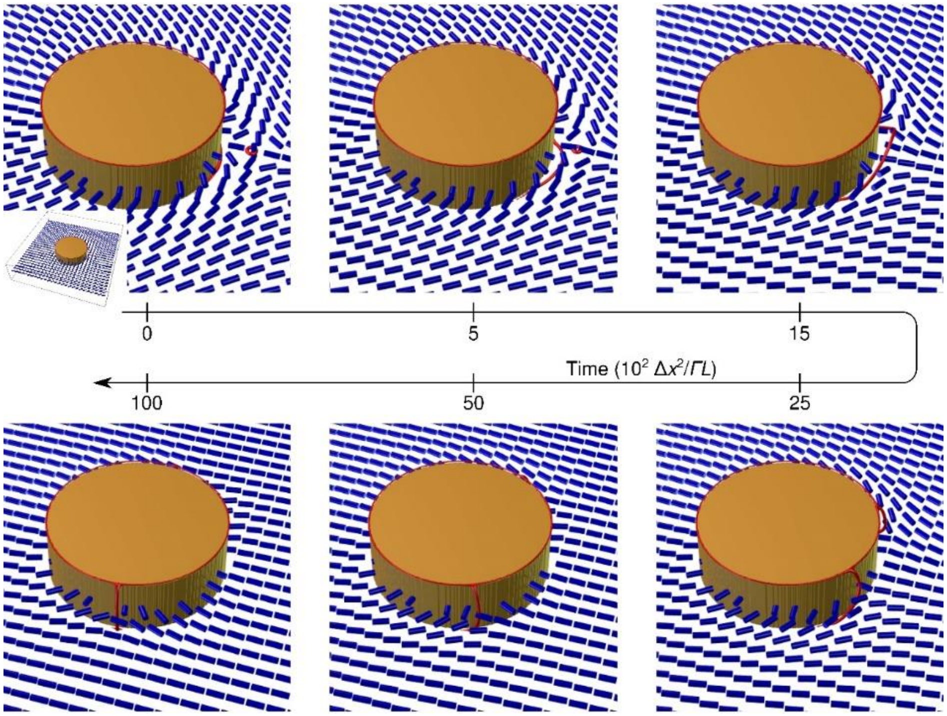

Fig. S5. Time evolution from the dipolar to the quadrupolar configuration on a static hybrid disk colloid. The initial configuration is a dipolar director field with a point defect not in contact with the disk surface. Inset shows the disk position and dimension inside the numerical simulation box. In time, the point defect comes in close proximity to the defect line protruding from the disk bottom edge $(t = 500\Delta x^2 / \Gamma L)$, and eventually they merge $(t = 1500\Delta x^2 / \Gamma L)$. The newly formed defect line then gets pinned to the top edge $(t = 2500\Delta x^2 / \Gamma L)$, forming two line segments that gradually move away from each other, leading to a stable quadrupolar director field configuration.

The static defect stable defect configuration in experiment is a dipolar loop adjacent to the colloid. We perform fluorescence confocal microscopy experiments to confirm its structure. Details are in the main text.

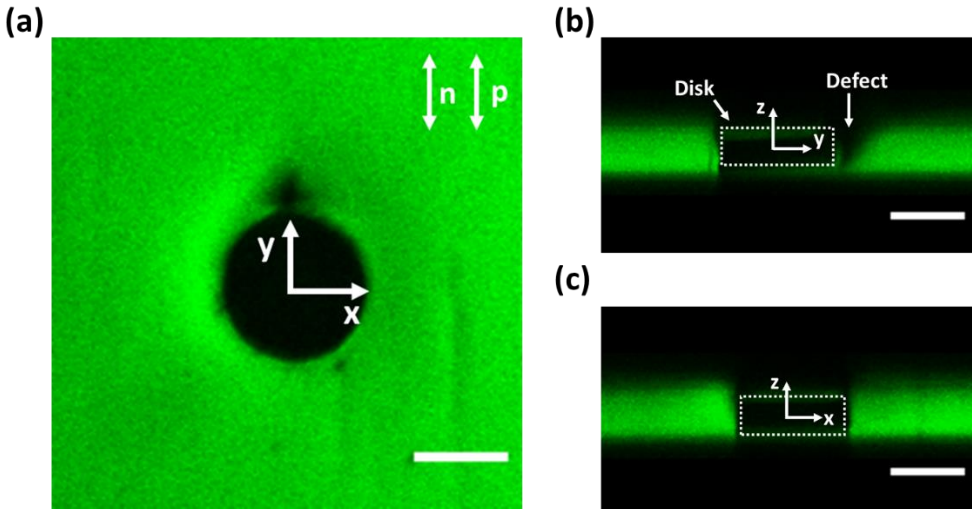

Fig. S6. Characterization of static defect structure. Fluorescent confocal polarizing microscopy (FCPM) images of a disk colloid with dipolar defect shown in (a) xy-plane, (b) yz-plane and (c) xz-plane. Scale bars are $50\mu m$.

S3. Calculation of the tilting angle of the disk from its projected area

The projected area of a disk with finite thickness is $A = \pi R_D^{\,2} \cos\alpha + 2R_D H_D \sin\alpha$, where R_D is the radius of the disk, H_D is the disk thickness and α is the angle made by the disk with respect to the x axis in its tilted state. The ratio $\frac{A}{A_0} = \cos\alpha + \frac{2H_D}{\pi R_D}\sin\alpha$. For our disk with $2R_D = 75\mu mn, H_D = 25\mu m$, $\frac{A}{A_0} = \cos\alpha + \frac{1.33}{\pi}\sin\alpha$. The maximum tilting angle for our disk for the sandwich cell of gap $H_{Gap} = 50$ μm is $\alpha_{\max} \sim 20°$ calculated form geometric confinement. Since the expression for $\frac{A}{A_0}$ increases monotonically for $0 \le \alpha \le \alpha_{\max}$, the value for α can be inferred by tracking the projected area of the disk. For a disk swimmer in a cell of $H_{Gap} = 50$ μm , we calculated the tiling angle of the disk in its most tilted state from the maximum $\frac{A}{A_0}$, as shown in Fig. 2B in the main text, to be $\sim 12°$

S4 Swimmers' translation does not depend on the strength of the applied field

We performed control experiments to dispel concerns that the translation might result from field gradients in the domain by studying swimmer motion for fields of differing amplitudes. Details are in the main text.

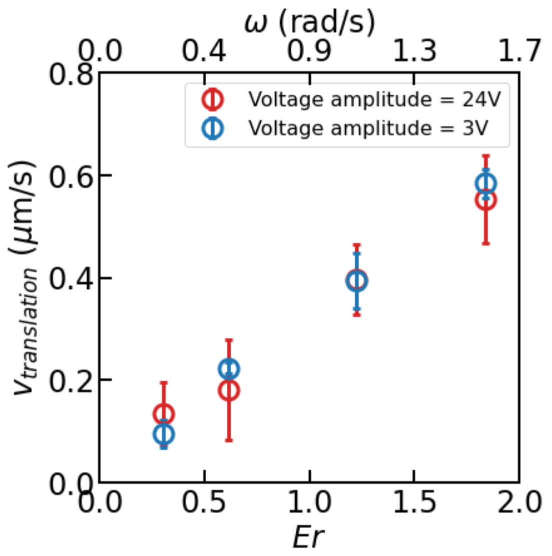

Fig. S7. Swimming speed does not depend on the strength of the rotating field. Translational speed of topological swimmers as a function of Ericksen number under different field amplitudes of 3V and 24V.

S5. Experiments to confirm the roles of elasticity, defect pinning and broken symmetry in the swimmers' translation

We have performed experiments to confirm the roles of elasticity, defect pinning and broken symmetry.

Rotation of disk colloids in the isotropic phase of 5CB To examine the importance of NLC elasticity in the observed translation of the rotated disk, the same colloids were rotated in the isotropic phase of 5CB. A planar cell filled with the disk colloid suspension was sealed on all sides using epoxy resin and submerged in a hot water bath with temperature higher than the phase transition temperature of 5CB. The 5CB in the sealed cell was melted into the isotropic state and the water bath ensured that any possible drift from temperature gradients was minimized. An external field was applied to rotate the disk in the isotropic phase of 5CB. In comparison to disks rotated in the nematic phase, the translational velocity was attenuated by more than an order of magnitude (see MovieS3). For example, the apparent velocities of disk colloids rotating in isotropic and nematic phase of 5CB are $0.025\mu m/s$ and $0.94\mu m/s$ respectively under the same external field with period $T=20s$. Notably, the velocity is decreased even though the viscosity is reduced; the viscosity of 5CB decreases with temperature, and is lower in the isotropic phase than in the nematic phase.

Controlled rotation of spherical colloids in the nematic phase of 5CB

To demonstrate the importance of defect line pinning in the observed translation, $2a=8.74\mu m$ ferromagnetic spherical colloids (Spherotech, Inc.) were treated with DMOAP to impose homeotropic anchoring on the surface before being dispersed in 5CB. The colloidal suspension was introduced into a planar cell of thickness $H\sim 30\mu m$ and the cell was placed under an external rotating magnetic field with period $T=6s$. Spherical colloids rotated with the field with their hedgehog defect oscillating around the initial equilibrium as shown in supporting Movie S3.

During this process, no translation was observed. Notably, the defect in this example was not pinned and did not stretch as the colloid rotated. Rather, the companion defect moved freely above the colloid.

Comparison of translation of rotating disk colloids with differing defect configurations
We have compared the translation of swimmers with dipolar defects to that of disk colloids with defects in quadrupolar simulations.

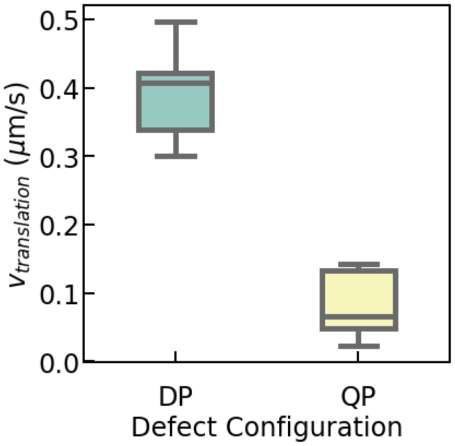

Fig. S8. Broken symmetry is essential for topological swimmers. Comparison of translational speeds of topological swimmers with dipolar (DP) and quadrupolar (QP) defect under the same external rotating magnetic field of T=60s.

S6. Simulation of defect dynamics around rotated disk colloid

Defect motion is simulated for the quadrupolar nematic structure using the full backcoupling of the nematic orientation to the flow field by including the nematic stress tensor in the Beris-Edwards formulation

$$\sigma_{ij}^{\text{nematic}} = -\zeta H_{ik}\left(Q_{kj} + \frac{\delta_{kj}}{3}\right) - \zeta\left(Q_{ik} + \frac{\delta_{ik}}{3}\right)H_{kj} +$$

$$2\zeta\left(Q_{ij} + \frac{\delta_{ij}}{3}\right)Q_{kl}H_{kl} + Q_{ik}H_{kj} - H_{ik}Q_{kj} - \frac{\delta F}{\delta\partial_j Q_{kl}}\partial_i Q_{kl} + f_{\text{LdG}}\delta_{ij}$$

in the Navier-Stokes equation, where f is the free energy density from Eq. S4. The simulation was performed for the value of the rotational viscosity parameter of $\Gamma = 1.4/\eta$, where η is the isotropic viscosity. A snapshot of the relevant fields is shown after 20000 timesteps. The backflow is generated by the stresses imposed on the fluid due to nematic alignment.

In Fig. S9, A nematic stress tensor in the Beris-Edwards formulation is adopted to explicitly compare the simulations with and without backflow. As the flow field is imposed by the rotating surface of the disk, the changes in the velocity field are small in close proximity to the disk surface but reach up to ~8% further away from the disk and result in small changes of the defect positions (Fig. S9d). Furthermore, the changes in the flow field are distinctly nonhomogeneous due to nonhomogeneous ordering of the nematic around the defects.

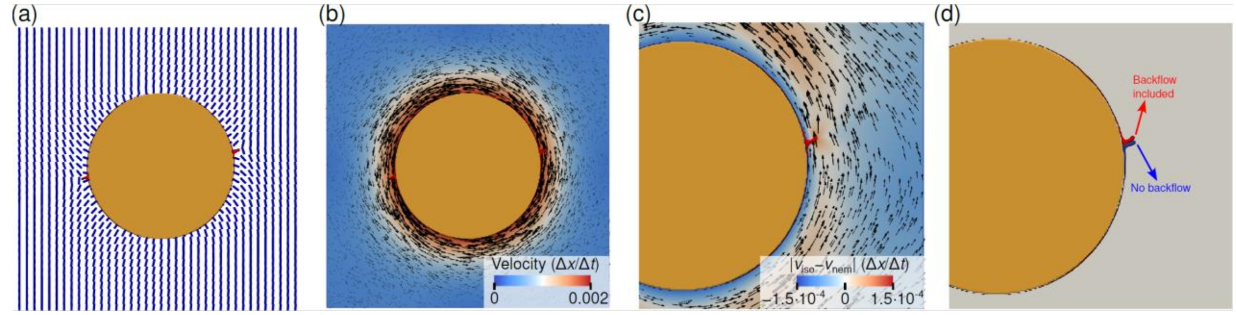

Fig. S9. Role of the full hydrodynamic solution. (a) Director field (blue rods) and the degree of order (red isosurface at $S = 0.4$) for the full hydrodynamic solution. (b) Flow-field for the full hydrodynamic solution. The velocity boundary condition is set at the surface of the rotating disk with the maximal velocity of $0.002\Delta x / \Delta t$. (c) Velocity difference between the isotropic viscosity (i.e. no backflow) solution and the fully coupled solution with the nematic stress tensor. The velocity difference is around 8% and is distinctly inhomogeneous around the region of the topological defect. (d) Using the fully hydrodynamic coupling, we observe little difference in the defect dynamics. The nematic defects for the coupled solution (red) and no-backflow solution (blue) are close to each other.

For disk colloids with dipolar defect in the decoupled simulation, rotation of the disk displaces two defect lines along the flow field. The dynamics of the transformation is shown in MovieS5.

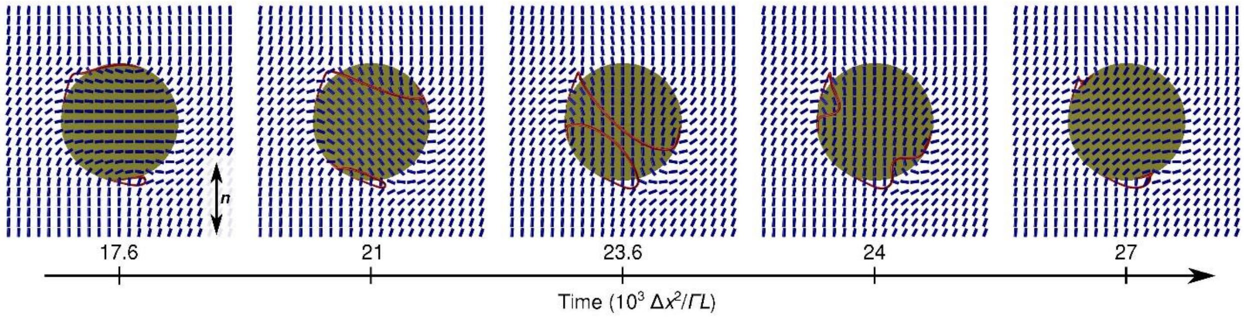

Fig. S10. Defect sweeping motion in simulation. The timeline shows a period of defect motion as seen in MovieS5. Disk is viewed from the bottom directly towards the planar surface. Surface anchoring profile rotates with the disk and generates a sweeping motion of the defect lines across the disk surface.

S7. Estimate of hydrodynamic force in lubrication limit during the tilting and flattening of the disk

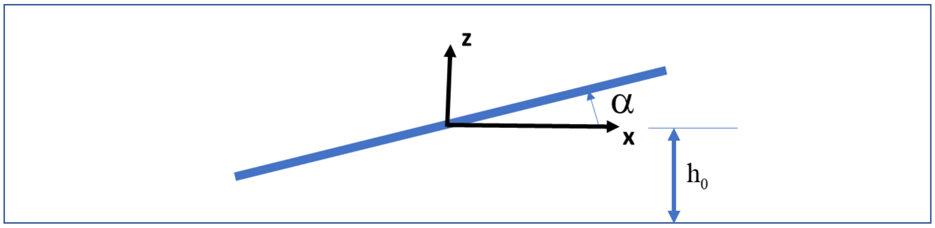

Fig. S11. Geometry defining the coordinates used in the calculation of the lubrication force and the resulting translational velocity. The disk tilts in the xz-plane with a tilting angle α. The thickness of the film between the bottom surface of the disk and the bottom bounding plate when the disk is flat is defined as h_0.

We approximate the disk as a plate of total length and width given by $L = W = 2a.$ The plate is tilted with angle $\alpha(t)$ with small slope $\alpha \sim \varepsilon = \frac{h_0}{a}$. We consider the fluid in the thin film between the plate and the wall. We derive the leading order force in the -x direction that arises for the plate in a tilted state. Let $z = h(x, \alpha(t))$ locate the disk's bottom surface. For small slopes, the liquid under the plate is defined by $0 \le z \le h$ and $-a \le x \le a.$ Let the height of the disk's bottom surface be defined $h = h_0 + x\tan\alpha \approx h_0 + x\alpha$, with minimum and maximum values of $h_{\min} = h_0 - a\sin\alpha \approx h_0 - a\alpha$ and $h_{\max} = h_0 + a\sin\alpha \approx h_0 + a\alpha$, respectively. The rate of change of the disk's surface is given by $\frac{\partial h}{\partial t} = x\dot{\alpha}\sec^2\alpha \approx x\dot{\alpha}$. Assuming that $\alpha \sim \varepsilon = \frac{h_0}{a} << 1$ and scaling $z \sim h_0$ and $x \sim a$, Stokes equations for motion in the thin film reduce to the lubrication equations:

$$\frac{dp}{dx} = \eta\frac{\partial^2 u_x}{\partial z^2}; \quad \frac{\partial u_x}{\partial x} + \frac{\partial u_z}{\partial z} = 0$$

The hinging motion of the tilting plate requires $u_x(h) = -x\dot{\alpha}\alpha.$ No slip at the bottom bounding plate requires $u_x(z = -h_0) = 0$. The lubrication equation and boundary conditions allow the velocity in the x direction to be determined.

$$u_x = \frac{1}{\eta}\frac{dp}{dx}\frac{(z^2 - h_0^{\,2})}{2} + \left(\frac{-x\dot{\alpha}\alpha}{(h + h_0)} - \frac{1}{\eta}\frac{dp}{dx}\frac{(h - h_0)}{2}\right)(z + h_0).$$

The plate motion generates flow according to the kinematic condition $u_z(h) \approx \frac{\partial h}{\partial t} + u_x(h)\alpha$.

Using the equation of continuity, the kinematic condition, and the no-slip condition at the bottom plate $u_z(z = -h_0) = 0$, we find the thin film equation:

$$\left(\frac{1}{12\eta}\frac{d}{dx}\left(\frac{dp}{dx}(h + h_0)^3\right)\right) = \dot{\alpha}(x + \alpha h_0)$$

This expression can be integrated to find the leading order stresses generated in the thin liquid film by the plate motion.

$$\frac{1}{12\eta\dot{\alpha}}(p(x) - p_0) = \frac{1}{2\alpha^3}\left[\ln\frac{(h(x) + h_0)}{(h_{\min} + h_0)} - 4h_0\left[\frac{1}{(h_{\min} + h_0)} - \frac{1}{(h(x) + h_0)}\right] + 2h_0^{\,2}\left[\frac{1}{(h_{\min} + h_0)^2} - \frac{1}{(h(x) + h_0)^2}\right]\right] +$$

$$+\tilde{c}_3\left[\frac{h_0^{\,2}}{(h_{\min} + h_0)^2} - \frac{h_0^{\,2}}{(h(x) + h_0)^2}\right]$$

where

$$\tilde{c}_3 == \frac{-1}{2\alpha^3}\left\{\frac{(h_{\max} + h_0)^2(h_{\min} + h_0)^2}{h_0^{\,4}}\right\}\left[\left[\frac{(h_{\min} + h_0)^2(h_{\max} + h_0)^2}{h_0^{\,3}[h_{\max} + h_{mn} + 2h_0])}\right]\frac{h_0}{(h_{\max} - h_{mn})}\ln\frac{(h_{\max} + h_0)}{(h_{\min} + h_0)} - 4\left[\frac{(h_{\min} + h_0)(h_{\max} + h_0)}{[h_{\max} + h_{mn} + 2h_0]}\right] + 2\right].$$

This stress can be used to evaluate the force in the x direction that acts on the tilted plate:

$$\mathrm{F} = -2a \int_{-a}^{a} \left(p(x) - p_0 \right)(\alpha) dx \cdot$$

The resulting force is

$$\frac{\mathrm{F}}{\frac{2}{\alpha^3}\eta\dot{\alpha}\; h_0 a} = -6\left(\frac{(h_{\max} + h_0)}{h_0} \ln\frac{(h_{\max} + h_0)}{(h_{\min} + h_0)} - \frac{(h_{\max} - h_{\min})}{h_0} \right)$$

$$+\; 2\left(\frac{(h_{\max} - h_{\min})}{(h_{\min} + h_0)} - \ln\frac{(h_{\max} + h_0)}{(h_{\min} + h_0)} \right) - 12\left(1 + \tilde{c}_3\alpha^3\right)\frac{(h_{\max} - h_{\min})}{h_0}\left(\frac{h_0^{\,2}}{(h_{\min} + h_0)^2} - \frac{h_0^{\,2}}{(h_{\min} + h_0)(h_{\max} + h_0)} \right)$$

The expression for the force can be recast in terms of $\rho = \alpha\varepsilon^{-1}$:

$$\mathrm{F} = \frac{-1}{\alpha^3}\left(12ah_0\; \eta\dot{\alpha}\right)\left(\ln\frac{(2+\rho)}{(2-\rho)}\; \frac{1}{2}\left(12 - \rho^2\right) - 6\rho \right)$$

This expression is valid for angle α comparable to the small parameter $\varepsilon = \frac{h_0}{L}$. To capture the limit of fixed $\varepsilon = \frac{h_0}{L}$ and small $\alpha << \varepsilon$, the scaling must be re-formulated, e.g. as a squeezing flow with very weak tilt. The divergence in small α would then be relieved.

The disk periodically tilts and flattens as the disclination line performs a swim stroke. On the top face of the disk, where the defect performing the swim stroke is absent, the disk does not experience a net force due to the scallop theorem since the tilting and flattening are completely reversible, generating no net displacement. However, below the disk, the defect sweeps over the disk's bottom face, causing it to tilt, and is absent during the flattening process. The resulting difference in viscosity in the thin gap with and without the defect avoids the constraints of the scallop theorem, and results in a net hydrodynamic force on the disk.

We consider one cycle of disk tilting and flattening generated by the defect's sweeping motion; the tilting and flattening events each occur over time $0.3T$ at a roughly constant rate $|\dot{\alpha}| = \alpha_{\max} / (0.3T)$. The disk tilts with $\dot{\alpha} > 0$ in the presence of the defect between the disk's surface and the bottom confining wall. The disk flattens with $\dot{\alpha} < 0$ after the defect has left this thin gap. When the defect is present, the film has an effective viscosity η_{defect}. When the defect is absent, the film has the viscosity representative of bulk nematic η. This allows the velocity during the sweeping event to be estimated:

$$v \approx \frac{1}{C_D}\left(F_{defect} + F_{no-defect}\right) = \left(\frac{a\eta}{C_D}\right)\frac{6\,h_0\;|\dot{\alpha}|}{\alpha^3}\left(\frac{\Delta\eta}{\eta}\right)\left(\ln\frac{(2+\rho)}{(2-\rho)}\; \left(12 - \rho^{\,2}\right) - 12\rho \right)$$

This expression is evaluated for typical material parameters in the main text.

S8. Disks roughness

Disk roughness is implicated in stochastic nature of defect pinning and depinning in the main text. The roughness is characterized by AFM.

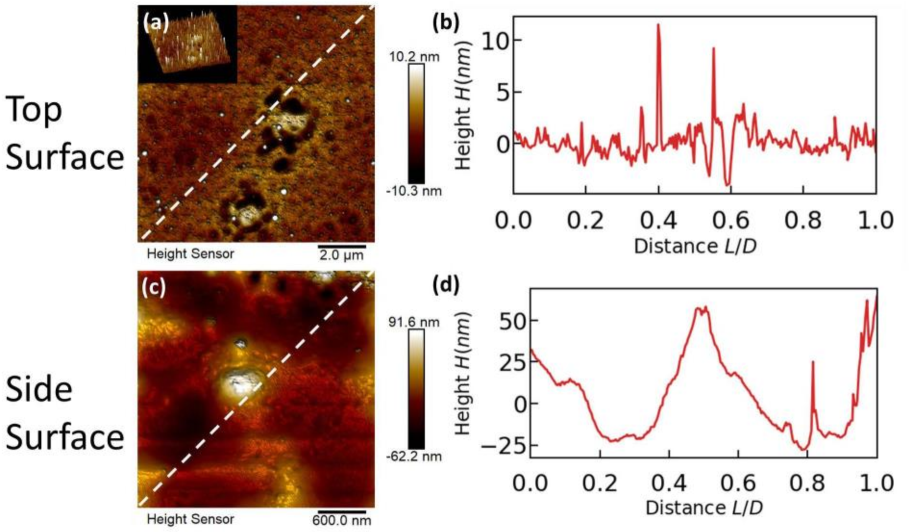

Fig. S12. Roughness of the disk colloid's top and side surfaces. (a) and (c) AFM images of a 10 µm by 10 µm area on the top surface of the disk and a 3 µm by 3 µm area on the side surface of the disk, respectively. Scale bars are 2 µm and 600 nm. (b) and (d) indicate the height profile along the white dash diagonals in (a) and (c). Note difference in scale in (b) and (d).

S9. Confinement and swimmers' translational speed

We have probed the velocities of disk swimmers as a function of confinement. The results are discussed in the main text.

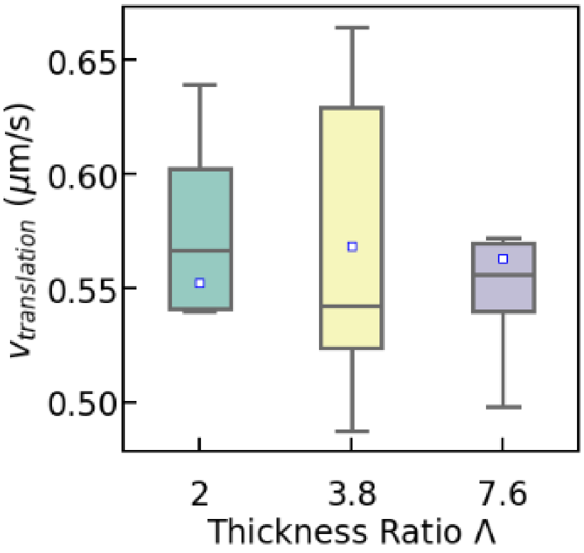

Fig. S13.Translational speeds for swimmers at fixed Er under different confinement. The disks move with similar translation speeds, indicated in the box plots in which the squares indicate the means and the lines within the

box indicate the medians of multiple observations under three different confinements Λ =2, 3.8 and 7.6, respectively.

S10 Co-rotation of dynamic dimer pairs formed by swimmers of opposite defect polarity

We tracked the separation distance between two disk swimmers of opposite polarity as they form anti-parallel dimers and co-rotate. For stable dynamic dimers, the maximum separation distance increases during a periodic encounter as *Er* decreases due to weaker attractive defect-defect interactions for both Λ = 2 and 3.8 cells (Figs. S13a and S13b). In some cases, dynamic dimers become unstable, and the two swimmers moves away from each other as shown the main text and Fig. S13c.

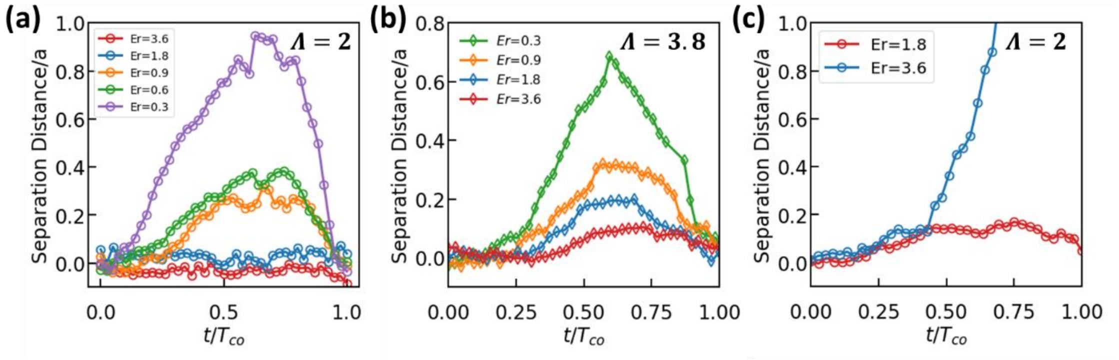

Fig. S14. Co-rotation of dynamic dimer pairs formed by two anti-parallel swimmers. Separation distance (center-to-center distance subtracted by two radii) during one co-rotation period for stable dynamic dimers in cells with (a) Λ=2 and (b) Λ=3.8. (c) The separation distance of a stable dynamic dimer (red) and an unstable dynamic dimer (blue) in a cell with Λ=2. In order to show the motion of the stable and unstable dynamic dimers on a single graph, the data for stable dynamic dimers are normalized by the co-rotation period of the dimer pair, while the data for the unstable dynamic dimers, which migrated apart, is normalized by the time required for the disks to be separated by two disk radii.

S11 Co-migration of two colloids with same defect polarity without dimerization

Two rotating disk colloids with the same polarity co-migrated without dimerization or defect entanglement below a threshold Ericksen number. The pair co-migrated in a head-to-tail fashion and moved along a well-defined direction similar to the direction selected by individual swimmers as shown in supporting Movie S8. However, the swimming velocities of the co-migrating pair were much lower than individual colloids under the same external field.

Movie S1

Topological defect-propelled swimming. Rotation of a disk colloid in the nematic phase of 5CB showing the swim stroke of the far-from-equilibrium defect as it sweeps across the disk's lower surface. This is the basis of the swimming of the disk colloid. In this experiment, the disk is rotated by a counterclockwise rotating external field of period $T = 40s$. The black double headed arrow indicates the orientation of the far-field director. The video is 10 times faster than real time and the scale bar is $50\mu m$. The corresponding process is shown in Fig. 1D and illustrated in Fig. 1E in the main text.

Movie S2.

Trajectory planning. **a**. Two disk swimmers follow V-shaped trajectories under a rotating external field of period $T = 6s$. The sense of rotation of the external magnetic field is switched from counterclockwise to clockwise at t=9.2s in the video. The video is 20 times faster than real time. **b**. A disk swimmer following a curved trajectory under a counter clockwise rotating external field with changing periods from $T_1 = 4s$ to $T_2 = 12s$ to $T_3 = 36s$ at $t = 1s$ and $t = 4s$ in the video. The video is 40 times faster than real time. The black double headed arrow indicates the far-field director and the scale bar are $100\mu m$. The corresponding process is illustrated in Fig. 4A and Fig. 4D in the main text, respectively.

Movie S3.

Control experiments. **a**. Swimming is absent in the isotropic phase. Rotation of a disk colloid in the isotropic phase of 5CB under a rotating external field of period $T = 20s$. The video is 10 times faster than real time and the scale bar is $50\mu m$. **b**. Swimming is absent for colloids that lack pinned defects. Rotation of a ferromagnetic spherical colloid with diameter $2a = 8.74\mu m$ with hedgehog companion defect in a planar cell filled with 5CB under a counterclockwise rotating external field of period $T = 6s$. **c**. Swimming is absent for defects that lack broken symmetry. Rotation of a disk colloid with quadrupolar defect in the nematic phase of 5CB under a counterclockwise rotating external field of period $T = 60s$. The defect traveled along the edge of the disk and no translation was observed during rotation due to the lack of broken symmetry. The video is 20 times faster than real time and the scale bar is $50\mu m$. The black double headed arrows indicate the far-field director.

Movie S4.

Quadrupolar nematic structure deformed by the flow field of the rotating disk from numerical simulation. Two defect lines are initially at the far left and far right edge of the disk. As the disk starts to rotate, defect lines move along the flow field until they reach a static position in which the elastic force on the defect lines counterbalances the force due to the velocity field. The simulation was performed at $Er = 1.22$, final structure of the velocity and the director field is shown also in Fig. S9.

Movie S5.

Defect sweeping motion of a dipolar structure and a rotating disk from numerical simulation. In the simulation, the nematic rotational viscosity parameter Γ_{surf} is reduced by a factor of 10^4 at the bottom surface. This causes a frustrated director field when the bottom disk surface is rotated for more than $\pi / 2$ relative to the fixed director field at the bottom plane of the simulation box.

The frustration is relieved by a sweeping motion of the disclination line across the disk bottom surface. Notice that the sweeping motion occurs every π turn rotation of the bottom surface. In experiments, pinning of defect lines on the defect edge leads to a sweeping motion on every $\frac{\pi}{2}$ turn. Simulation is performed at $Er = 1.22$ and lasts for $5\cdot 10^3 \Delta x^2 / (\Gamma L)$.

Movie S6.

Rotation of single swimmers in weakly confined cell. The video shows single swimmers rotating in weakly confined cells ($\Lambda = 6$ and $\Lambda = 7.6$) under an external magnetic field of T=1200s. The disk in the $\Lambda = 6$ cell is in a counter clockwise rotating external field; the disk in the $\Lambda = 7.6$ cell is in a clockwise rotating external field. The video is 100 times faster than real time. The black double headed arrows indicate the far-field director. The scale bars are $50\mu m$. The corresponding process is illustrated in Fig. 5C and discussed in the main text.

Movie S7.

Antiparallel dimer co-rotation. Co-rotation of a dimer formed by two disk colloids with opposite defect polarity under a clockwise rotating external field of period: **a**. $T = 80s$. **b**. $T = 40s$ and **c**. $T = 20s$. The video is 40 times faster than real time for **a** and 10 times faster than real time for **b** and **c**. The black double headed arrows indicate the far-field director and scale bars are $50\mu m$. The corresponding process is illustrated in Fig. 6B and discussed in the main text.

Movie S8.

Dynamic Interaction between multiple simmers. Dynamic interaction between topological flagella of multiple swimmers under a counterclockwise rotating external field of period $T = 6s$. The black double headed arrow indicates the far-field director. The video is 20 times faster than real time and the scale bar is $50\mu m$. The formation of the unstable trimer demonstrated in Fig. 7 in the main text is shown in the lower left region of the video.

Movie S9.

Co-migration of two swimmer with the same defect polarity without co-rotation. Co-migration of two disk colloids with the same defect polarity under a counterclockwise rotating external field of period $T = 6s$. The black double headed arrow indicates the far-field director. The video is 20 times faster than real time and the scale bar is $50\mu m$.

Movie S10.

Co-rotation and translation of two swimmer with the same defect polarity. Co-rotation and translation of a dimer formed by two disk colloids with the same defect polarity under a counterclockwise rotating external field of period $T = 4s$. The black double headed arrow indicates the far-field director. The video is 20 times faster than real time and the scale bar is $50\mu m$. The corresponding process is illustrated in Fig. 6D in the main text.

Movie S11.

Dipole to quadrupole transition in a numerical simulation. Point defect in the dipolar initial structure approaches the disk and forms two disclination lines, each pinned at the bottom and at

the top edge of the disk. Disclination lines move along the edges of the disk until they finally form a quadrupolar structure. The duration of the movie is $1.8\cdot 10^3 \Delta x^2 / (\Gamma L)$ in simulation units.
